# Ultrasound quadriceps muscle thickness is variably associated with frailty in haemodialysis recipients

**DOI:** 10.1186/s12882-022-03043-8

**Published:** 2023-01-18

**Authors:** Benjamin M. Anderson, Daisy V. Wilson, Muhammad Qasim, Gonzalo Correa, Felicity Evison, Suzy Gallier, Charles J. Ferro, Thomas A. Jackson, Adnan Sharif

**Affiliations:** 1 Department of Nephrology and Transplantation, Queen Elizabeth Hospital, Birmingham, B15 2WB UK; 2 Institute of Inflammation and Ageing, University of Birmingham, Birmingham, UK; 3 Department of Healthcare for Older People, Queen Elizabeth Hospital, Birmingham, UK; 4 Institute of Immunology and Immunotherapy, University of Birmingham, Birmingham, UK; 5 Hospital del Salvador, Santiago, Chile; 6 Department of Health Informatics, Queen Elizabeth Hospital, Birmingham, UK; 7 PIONEER: HDR-UK hub in Acute Care, Edgbaston, Birmingham, UK; 8 Institute of Cardiovascular Sciences, University of Birmingham, Birmingham, UK

**Keywords:** Sarcopenia, Frailty, Ultrasound, Epidemiology

## Abstract

**Background:**

Ultrasonographic quantitation of quadriceps muscle mass is increasingly used for assessment of sarcopenia, but its relationship with frailty in haemodialysis recipients is not known. This study explores the relationship between ultrasound-derived bilateral anterior thigh thickness (BATT), sarcopenia, and frailty by common frailty tools (Frailty Phenotype [FP], Frailty Index [FI], Edmonton Frailty [EFS], and Clinical Frailty Scale [CFS]).

**Methods:**

This was an exploratory analysis of a subgroup of adult prevalent (≥3 months) haemodialysis recipients deeply phenotyped for frailty. Ultrasound assessment of BATT was obtained with participants at an angle of ≤45°, with legs outstretched and knees resting at 10°-20°, according to an established protocol. Associations with frailty were explored via both linear and logistic regressions for BATT, Low Muscle Mass (LMM), and sarcopenia with stepwise adjustment for a priori covariables.

**Results:**

In total 223 study participants had ultrasound measurements. Frailty ranged from 34% for FP to 58% for FI. BATT was associated with increasing frailty on simple linear regression by all frailty tools, but lost significance on addition of covariables. Upon dichotomising frailty tools into Frail/Not Frail, BATT was associated with frailty by all tools on univariable analyses, but only retained association for EFS on the fully adjusted model (OR 0.97, 95% C.I. 0.94–1.00, *P* = 0.05).

**Conclusions:**

Ultrasound measures of quadriceps thickness is variably associated with frailty in prevalent haemodialysis recipients, dependent upon the frailty tool used, but not independent of other variables. Further work is required to establish the added value of sarcopenia measurement in frail haemodialysis patients.

**Trial registration:**

Clinicaltrials.gov: NCT03071107 registered 06/03/2017.

**Supplementary Information:**

The online version contains supplementary material available at 10.1186/s12882-022-03043-8.

## Introduction

Frailty is a syndrome of increased vulnerability to poor resolution of homeostasis after a stressor event [1], associated with negative outcomes including mortality, hospitalisation and disability [2]. The gold standard in frailty diagnosis and treatment is the Comprehensive Geriatric Assessment (CGA) [3]. This is rarely used in clinical and/or research practice due to cost and logistical barriers; instead, a variety of frailty diagnostic and screening tools have been proposed. These range from the Frailty Phenotype (FP) [4], which focusses upon physical aspects of frailty, to the Frailty Index (FI) [5, 6], and Edmonton Frail Scale (EFS) [7], which conceptualise frailty as a multidimensional biopsychosocial syndrome. The Clinical Frailty Scale (CFS) [8], is a brief frailty tool derived from and validated against the FI. Previous work within the FITNESS study has shown that correlation is weak and agreement minimal between these scores in haemodialysis recipients [9], but all are associated with greater mortality [10].

Sarcopenia is a progressive and generalised disorder of skeletal muscle, defined by low muscle mass and low muscle strength, and is considered a contributor to the development of frailty [11]. Sarcopenia is under-recognised, in part due to difficulties confirming low muscle mass [11]. Gold standard assessments such as whole-body magnetic resonance imaging (MRI), computed tomography (CT) and dual-energy x-ray absorptiometry (DEXA) are expensive, time-consuming and onerous for patients [12]. Additional hospital visits for scans will be unacceptable for many haemodialysis recipients, given the already burdensome requirements of thrice-weekly dialysis. Despite these barriers, where DEXA has been used in research, prevalence of sarcopenia has been estimated at 37 and 40% in European and Japanese haemodialysis cohorts respectively [13, 14], and has been associated with hospitalisation and mortality in haemodialysis patients [13].

Ultrasound (US) is portable, often used on dialysis units for vascular access assessments, and has been validated for assessment of muscle size in older adults [15–17]. Recent work has suggested cut-offs for low muscle size – determined in the healthy young – according to ultrasound-derived bilateral anterior thigh thickness (BATT), a sum of the bilateral anterior-posterior depth of vastus intermedius and rectus femoris, which correlates with muscle strength and function [18]. Importantly, given some concerns in the literature regarding reproducibility of ultrasound measures [19], BATT shows high inter-rater reliability [18]. Ultrasound has also shown good agreement with CT measurements of mid-thigh quadriceps thickness and with bioimpedance [20, 21], and demonstrated little loss of precision by comparison to CT scanning in acute kidney injury patients [22]. Importantly, no significant differences have been identified in femoral muscle thickness before and after dialysis, indicating that fluid overload does not significantly influence results [23, 24]. Recent reports have shown that ultrasound-derived measures of quadriceps size are associated with both muscle function [25] and mortality [26] in haemodialysis recipients.

The combination of availability, cost, and simplicity therefore suggests that ultrasonographic assessment may be an attractive option for assessing sarcopenia in haemodialysis recipients but many questions remain unanswered. Firstly, bilateral anterior thigh thickness has not specifically been investigated in the setting of haemodialysis. Secondly, the relationship between US-derived muscle parameters and frailty has yet to be explored in haemodialysis patients. Therefore, the aims of this study were to; 1) explore the relationship between BATT with measures of muscle function, and 2) assess the relationship between muscle size, sarcopenia and frailty by common frailty tools both in the setting of prevalent haemodialysis patients.

## Methods

The FITNESS study follows a cohort multiple randomised controlled trial (cmRCT) design [27], the full protocol for which has been described in detail elsewhere [28]. The study protocol was approved by the South Birmingham Research Ethics Committee (Ref: 17/WM/0381). The study was conducted in accordance with the Declaration of Helsinki. This article describes analyses from the cohort study phase of FITNESS.

### Study setting

Patients were recruited from a single nephrology centre located in Birmingham, England, consisting of one in-hospital dialysis unit and ten private-provider satellite units distributed around the West Midlands. The service provides haemodialysis to patients in a mixture of urban and rural settings, with a diverse range of ethnic and socioeconomic groups. Eligible patients were identified by interrogation of hospital electronic patient records (EPR) and from discussion with clinicians at each dialysis unit. Informed consent was obtained from all participants after being given written and verbal information about the study, and given sufficient opportunity to consider the information before giving their consent to join the cohort study.

### Eligibility criteria

Inclusion criteria were adults aged 18 and over, anyone receiving regular haemodialysis for at least 3 months’ duration and the ability to give informed consent. The only exclusion criterion was inpatient care within 4-weeks of recruitment unless for vascular access purposes, to avoid confounding of baseline data with frailty secondary to recent hospitalisation.

### Baseline assessment

Baseline assessments of all study participants took place before and during one of their usual dialysis sessions. To negate the potential effect of the long break from dialysis upon frailty measurements, we avoided the first haemodialysis session after the weekend interval. Where participants dialysed twice weekly, the dialysis session after the shortest interval was chosen for baseline assessment.

Study participants completed a number of investigations, which are detailed in our methodology paper [28]. Briefly, prior to connection to dialysis, participants underwent a timed 4 m walk from standing and bilateral hand-grip strength via dynamometer (Takei Grip D, Takei Scientific Instruments Co. Ltd., Japan), alongside a Montreal Cognitive Assessment (MoCA [29]). Once dialysis started, patients were clinically interviewed, including a series of questionnaires including assessments of activities of daily living (ADL) disability, demography, social history, and frailty-specific questionnaires. PHQ-9, and GP Physical Activity (GPPAQ) [30] questionnaires were included. The Physical Activity Index was derived from the GPPAQ via a validated formula [30, 31]. Electronic Patient Records were interrogated for comorbidities, drug history, alongside dialysis vintage and adequacy, previous transplantation, and biochemical data. Self-reported change in health (henceforth “health change”) was obtained by asking the question “How has your health changed in the last year?” with potential responses of “Better” “The Same” or “Worse”. Determination of socio-economic deprivation was based upon the Index of Multiple Deprivation 2015 (IMD) [32], a multiple deprivation model calculated at the local level area, with 1 representing the most deprived and 5 the least deprived area respectively.

A full description of frailty tool components is contained in Supplementary Methods. Frailty by each tool was defined as: FP ≥3, FI ≥0.24, EFS ≥8, CFS ≥5.

As some participants had visual or manual dexterity impairment, abridged versions of MoCA were also permitted, by omitting executive function and naming sections for visually impaired, and the executive function section only for those lacking dexterity to draw [33].

### Muscle strength and function

Physical assessments took place immediately before connection to dialysis on the participants routine dialysis session. Grip strength was assessed using hand grip Dynamometer (Grip-D, Takei Scientific Instruments, Japan), with arm resting at the side of the patient with elbow in extension and wrist in the neutral resting position. A practice grip was taken with results discarded, then one summative grip on each hand, for which the participant was encouraged to give maximum effort. Both scores were noted, but the greater of the two scores taken for subsequent analysis. Adjusted hand-grip strength was calculated as fold change from the lowest 20% by gender and BMI in the original Fried frailty phenotype cohort [4].

Walking speed was measured over 4 m from a standing start; usual walking aids were permitted. If the participant was not able to complete the 4 m distance, no walking speed was calculated, and a deficit registered for this component of the relevant frailty scores.

### Ultrasonographic measurements

Following baseline frailty assessment, further verbal consent was sought for ultrasound assessment. Consenting participants had thickness of their anterior thigh muscles measured bilaterally, or unilaterally in the case of unilateral lower limb amputation. Ultrasonographic measurement took place during the participants’ regular dialysis session. Patients were positioned sitting at an angle of ≤45° with knees resting comfortably upon a cushion near the natural 10° to 20° resting position. Participants were instructed to relax during the examination. Scanning followed a protocol established in previous work [18], with subcutaneous tissue, vastus intermedius and rectus femoris depth all captured in a single transverse plane at anterior mid-thigh. This was defined as 50% of the measured distance between greater trochanter and lateral epicondyle of the femur. Images and depth measurements were obtained using Phillips Lumify L12–4 transducer via the Phillips Lumify app (Koninklijke Philips, Netherlands) on its factory musculoskeletal settings, with a power of − 0.3 dB and gain of 50. The bilateral anterior thigh thickness was calculated as the sum of bilateral rectus femoris and vastus intermedius anterior-posterior depth, or double the unilateral rectus femoris and vastus intermedius depth in instances of unilateral lower limb amputation, or of dialysis access (e.g. femoral line or arteriovenous graft) restricting adequate exposure of the area to be scanned. Thresholds for low muscle mass (LMM) were 38.53 m and 54.36 mm for females and males respectively, as per previous work in healthy volunteers [18]. Low grip strength was < 27 kg for males and < 16 kg for females; slow walking speed was set as < 0.8 ms^− 1^ [11]. Sarcopenia was defined as low muscle mass and low grip strength, severe sarcopenia was assigned when low muscle mass, low grip strength and slow walking speed were all present.

### Recruitment

As this was an exploratory study on a subgroup of FITNESS participants, no power calculation was applied to this part of the study.

### Statistics

Statistical analyses were performed using STATA (StataCorp. 2019. Stata Statistical Software: Release 17. College Station, TX: StataCorp LLC). Agreement was assessed with Cohen’s Kappa, and was rated as > 0.9 almost perfect agreement, 0.8–0.9 strong, 0.6–0.79 moderate, 0.4–0.59 weak, 0.21–0.39 minimal, and ≤ 0.2 no agreement.

Associations with continuous frailty scores were analysed using linear regression, having satisfied the linear assumption via visual comparison of observed versus Lowess fit lines on scatter plot and augmented component plus residual plots. Robust standard errors were specified to account for heteroscedasticity. Multicollinearity was excluded on all analyses by variance inflation factor < 10. Odds ratios for frailty were obtained by logistic regression.

Linear and logistic regressions were performed both unadjusted and adjusted for a priori covariables chosen for proven or suspected association with frailty. These were introduced in a stepwise manner; Model 1 included age, ethnicity, gender, education level, IMD quintile, self-reported social support (Yes/No), and haemodialysis vintage. Model 2 added to these self-reported health today (Euroqol 5D Visual Analogue Scale) [34], self-reported health change, PHQ-9 score, cognitive impairment, and Charlson Comorbidity Index (CKD omitted). Model 3 added self-reported slow walking speed (from GPPAQ), and use of walking aids (yes/no), which to avoid bias were chosen as – unlike grip strength and walking speed – they did not comprise a component of the frailty scores under investigation.

As statistical significance was lost for many frailty associations with stepwise addition of covariables, a sensitivity analysis was performed whereby the order of addition of covariables was reversed to explore the likelihood of type II error. Therefore, Model A included self-reported slow walking speed, and use of walking aids. Model B added self-reported health today [34], self-reported health change, PHQ-9 score, cognitive impairment, and Charlson Comorbidity Index (CKD omitted). Model C added age, ethnicity, gender, education level, IMD quintile, self-reported social support, and haemodialysis vintage.

Missing IMD quintiles were handled via a dummy variable. All other missing data were handled via listwise deletion, as < 1% of other data were missing. A *P* value < 0.05 was considered significant.

## Results

**Fig. 1:**
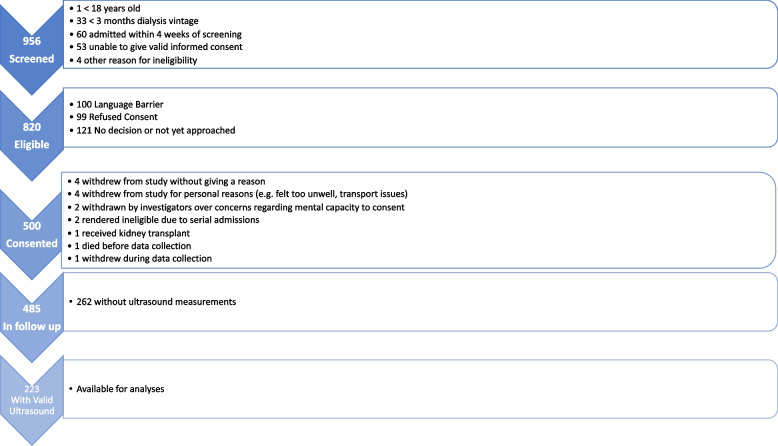
PRISMA flowchart of FITNESS study participation

Figure 1 shows a flowchart of recruitment to the FITNESS study. 485 participants underwent frailty assessment and entered follow-up, of which 223 had valid ultrasound measurements. Of those with valid US measurements, the median FP was 2 (IQR 1–3), FI 0.266 (IQR 0.148–0.430), EFS 7 (IQR 5–10, and CFS 4 (3–5). Table 1 shows key demographics; the percentage determined to be frail ranged from 34% for the FP to 58% for FI.

**Table 1:** Baseline characteristics of the FITNESS cohort and subgroup with valid ultrasound measurements

	n	% of cohort/IQR
Male	138	61.9
FP	76	34.1
FI	129	57.9
EFS	111	49.8
CFS	109	48.9
Albumin^a^	39	35–42
MoCA^a^	22	18–25
Age^a^	63	54–74
BMI^a^	27.0	23.4–32.1
Charlson Index^a,b^	5	3–6
HD Vintage (months)^a^	33	14–65
Kt/V^a^	1.58	1.37–1.82
Ethnicity		
*White*	136	61.0
*South Asian*	51	22.9
*Black*	32	14.4
*Other*	4	1.8
Smoking Status		
*Current*	33	14.8
*Ex*	67	30.0
*Never*	123	55.2
Active on transplant list?		
*No*	195	87.4
*Yes*	28	12.6
Employment Status		
*Employed*	32	14.4
*Unemployed*	74	33.2
*Retired*	117	52.5
Occupation^c^		
*Unskilled Manual*	92	43.4
*Skilled Manual*	32	15.1
*Clerical*	25	11.8
*Managerial*	31	14.6
*Professional*	32	15.1
Education level		
*High School*	154	69.1
*College/6th form*	47	21.1
*University*	22	9.9
Type of residence		
*Own Home*	219	98.2
*Warden-Controlled*	3	1.4
*Residential Home*	1	0.5
*Nursing Home*	0	0.0
Professional Carer Use?^d^		
*No*	209	94.1
*Yes*	13	5.9
Physical Activity Index		
*Inactive*	184	82.5
*Moderately Inactive*	18	8.1
*Moderately Active*	5	2.2
*Active*	16	7.2
IMD Quintile		
*1*	101	45.3
*2*	29	13.0
*3*	36	16.1
*4*	19	8.5
*5*	22	9.9
*Unknown*	16	7.2

*n* = 223. All values n and percentages except

^a^ = median and interquartile range

^b^ = CKD omitted

^c^ = or previous occupation if unemployed/retired

^d^ = if not in residential/nursing accommodation

### Prevalence of low muscle mass, and concordance with frailty

Low muscle mass was present in 119 participants (53.4%). Sarcopenia was identified in 71 participants (31.8%). Table 2 shows that concordance of both LMM and sarcopenia with frailty was poor regardless of frailty definition. Agreement between frailty and sarcopenia was minimal, Cohen’s Kappa was 0.381 for FP (*P* < 0.001), 0.219 for FI (*P* < 0.001), 0.245 for EFS (*P* < 0.001), and 0.240 for CFS (*P* < 0.001). Table 3 demonstrates that BATT was positively associated and LMM negatively associated with grip strength and walking speed on simple and multiple linear regressions.

**Table 2:** Concordance between frailty status with low muscle mass and sarcopenia

Frailty Tool	Frailty Status	Low Muscle Mass?		Sarcopenia?		Row Total (n)
		No	Yes	No	Yes	
FP	Not Frail	83	64	119	28	147
		56.5%	43.5%	81.0%	19.1%	
	Frail	21	55	33	43	76
		27.6%	72.4%	43.4%	56.6%	
FI	Not Frail	48	46	77	17	94
		51.1%	48.9%	81.9%	18.1%	
	Frail	56	73	75	54	129
		43.4%	56.6%	58.1%	41.9%	
EFS	Not Frail	61	51	90	22	112
		54.5%	45.5%	80.4%	19.6%	
	Frail	43	68	62	49	111
		38.7%	61.3%	55.9%	44.1%	
CFS	Not Frail	60	54	91	23	114
		52.6%	47.4%	79.8%	20.2%	
	Frail	44	65	61	48	109
		40.4%	59.6%	56.0%	44.0%

**Table 3:** Simple and multiple linear regression of Grip and Walking Speed associated with BATT and Low Muscle Mass

		Coef.	Lower 95% C.I.	Upper 95% C.I.	P
BATT	Grip				
	*Univariable*	0.29	0.21	0.37	< 0.001
	*Multivariable*	0.10	0.02	0.19	0.019
	Walk Speed				
	*Univariable*	0.007	0.004	0.010	< 0.001
	*Multivariable*	0.005	0.002	0.008	0.001
Low Muscle Mass	Grip				
	*Univariable*	−3.36	−5.99	−0.73	0.013
	*Multivariable*	−3.23	−5.65	−0.82	0.009
	Walk Speed				
	*Univariable*	−0.155	−0.244	−0.066	0.001
	*Multivariable*	−0.115	−0.202	−0.028	0.010

### Association between muscle size, sarcopenia, and frailty

**Fig. 2:**
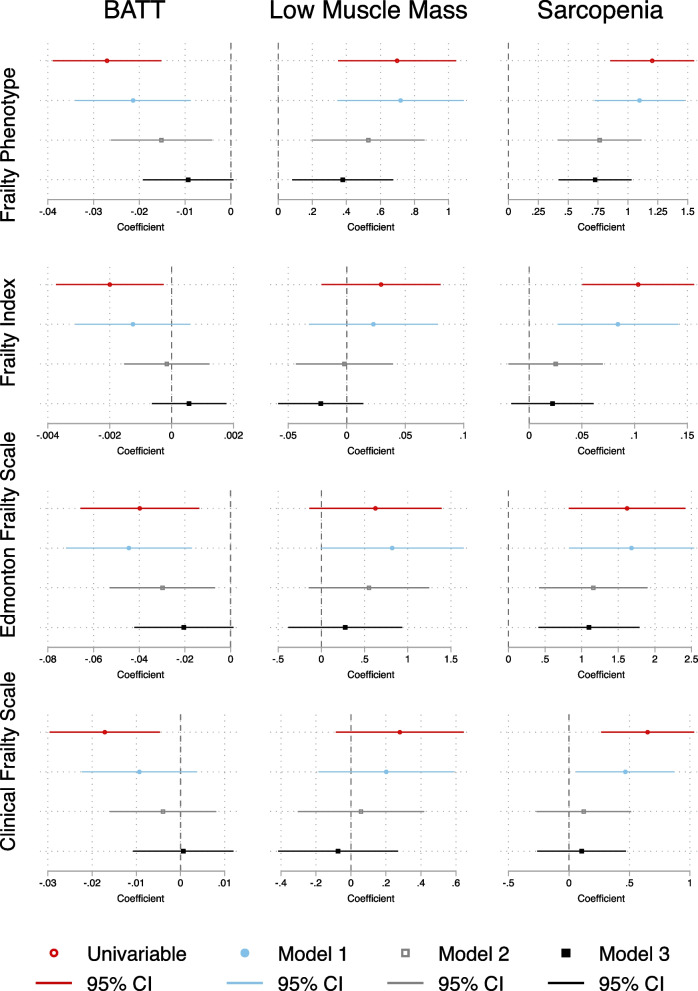
Composite of simple and multiple linear regression models of association between BATT, Low Muscle Mass, and Sarcopenia with Frailty

A composite forest plot of simple multiple linear regressions between each of BATT, LMM and sarcopenia with each frailty tool is shown in Fig. 2. It shows that increasing BATT was associated with lower scores on each frailty tool on simple linear regression. However, upon stepwise addition of covariables to the models, BATT lost its association with decreasing frailty by each tool. LMM was associated with increasing FP frailty on all linear regression models but was not significantly associated with any other frailty score. Sarcopenia was associated with increasing frailty on all models for the FP and EFS, but lost significance of association upon addition of Model 2 covariables for FI and CFS. All Model 3 results are detailed in Supplementary Tables 8, 9, 10, 11.

**Fig. 3:**
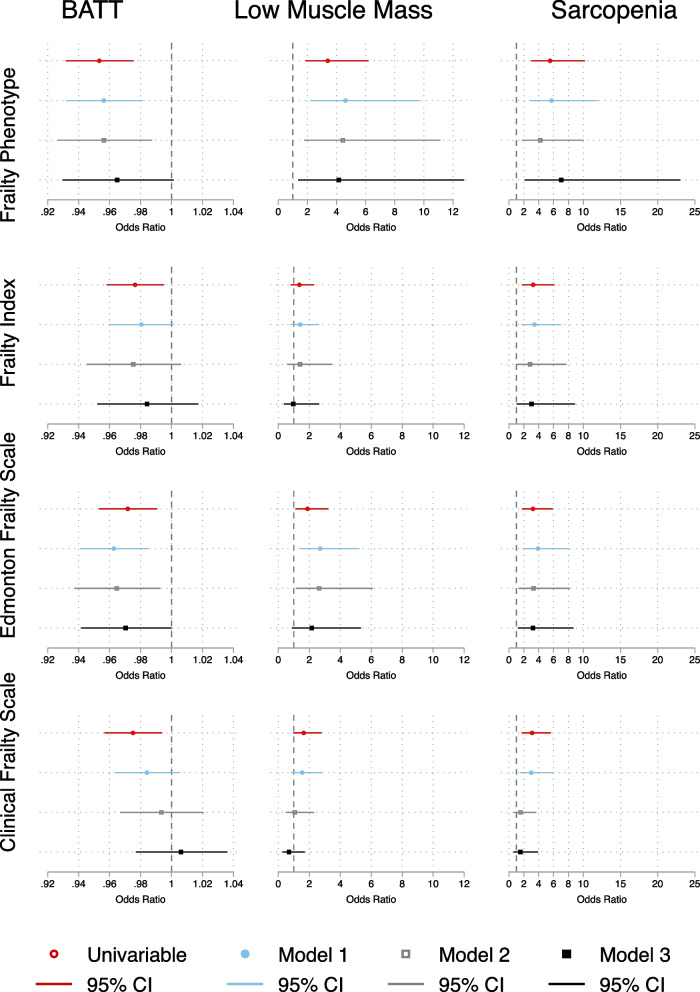
Composite of univariable and multivariable logistic regression models of association between BATT, Low Muscle Mass, and Sarcopenia with Frailty

A composite forest plot of odds ratios for frailty associated with BATT, LMM and sarcopenia is shown in Fig. 3. Increasing BATT was associated with lower odds of frailty for FP and EFS on univariable analyses and in Models 1 and 2 but lost the association on addition of surrogates of muscle function in Model 3. Neither frailty by FI nor CFS showed any significant associations with BATT on univariable or multivariable logistic regression.

A similar pattern was observed for LMM, which was associated with higher odds of FP frailty on all analyses, and for EFS frailty until addition of Model 3 covariables. It did not associate with FI or CFS frailty on any model. Sarcopenia was associated with increased odds of frailty on unadjusted and all adjusted models for FP, FI, and EFS, but lost significance for this association on Model 2 for CFS. All Model 3 results are shown in Supplementary Tables 8, 9, 10, 11.

### Sensitivity analyses

Supplementary Tables 12, 13 show that changing the order of covariable addition did not materially change the interpretation of results. The only exceptions were for linear and logistic regressions for association between FP and BATT; significant results were maintained until the final model on both forward and backward stepwise regression, suggesting the loss of statistical significance for Model 3 could be due to Type II error for the FP.

## Discussion

Ultrasonography has shown promise in assessing muscle size in frail older adults [15, 18], and in haemodialysis recipients [13, 14], but its relationship to frailty in haemodialysis is not known. Here we have shown in a large prevalent haemodialysis cohort that BATT associates with poorer grip strength and walking speed. Ultrasound-derived BATT, low muscle size [18], and sarcopenia that incorporates BATT criteria are associated with frailty in haemodialysis recipients, but consistency of these associations upon multivariable analyses are dependent upon the frailty measure used. The most persistent associations were seen with the FP, a primarily physically-focussed frailty score, whereas weakest associations were seen with CFS. Further work should include the assessment of long-term outcomes associated with BATT in haemodialysis, and to establish whether sarcopenia associates with negative outcomes in haemodialysis recipients when adjusted for frailty.

Ultrasound-derived quadriceps muscle mass has been validated against CT and BIA in haemodialysis recipients [20, 25], and is not affected by volume overload [23, 24]. Our findings corroborate and build-upon work from Matsuzawa and colleagues, who found that cross-sectional area of rectus femoris associated with grip strength and walking speed [25]. Associations of ultrasound-derived muscle mass and frailty in haemodialysis have to the best of our knowledge not been examined until now. Here we show that quadriceps muscle thickness is associated with frailty to a variable degree, and almost universally lost association with frailty on addition of surrogates of muscle function (self-reported walking speed and use of walking aids) to the models. Our finding that sarcopenia – the addition of low grip strength to low muscle mass – was more robustly associated with frailty is consistent with these results: indeed low grip strength is a component of the FP, FI (by our definition), and EFS. Whilst we must exercise caution when comparing across different regression models, it is interesting that the strength and reliability of association between muscle mass and frailty appear highest where the frailty tool most relies upon musculoskeletal constituents. The proportion of each frailty score ascribed to markers of muscle function ranges from FP (which almost exclusively relies upon markers of musculoskeletal function) at the highest, via the EFS, FI, to CFS (which does not include any direct assessment of muscle function) at the lowest. It is therefore unsurprising that the FP retained the strongest association with muscle mass and sarcopenia, but to our knowledge this is the first study to confirm this in haemodialysis recipients.

Despite sharing some mechanistic and clinical similarities, we must not conflate sarcopenia and frailty. Rather sarcopenia is considered a contributor to – but not a requirement of – frailty [11]. Reijnierse and colleagues reported that in older adults referred to a geriatric clinic, there was little concordance between sarcopenia and frailty [35], which we have corroborated in haemodialysis recipients here. The potential utility of sarcopenia assessment therefore depends upon additive risk stratification for important outcomes. Sabatino and colleagues found that distal vastus intermedius thickness – normalised for height – was independently associated with increased mortality in haemodialysis [26]. These results have yet to be repeated in other haemodialysis cohorts, but are in keeping with the association found between CT-derived thigh muscle mass and mortality in a Japanese haemodialysis cohort [36]. It remains to be established, however, whether the association with mortality is maintained when frailty is accounted for. If so, we can be confident that ultrasound measures of muscle mass offer prognostic detail that merit the logistical challenges. This question will be addressed as the FITNESS cohort matures.

Limitations of this study include the fact that the whole FITNESS cohort did not complete ultrasound assessment. The ultrasound scanner was not procured until after the first 193 participants were recruited, and a further 69 refused or were unable to be scanned. Scanning took place during haemodialysis sessions, in order to reduce the time commitment of each participant for the study. This may have resulted in the frailest being unable to participate in this aspect of the study due to concerns regarding mobility and adequate exposure for scanning. This potential discrepancy reduces the generalisability of our findings. The utility of quantifying muscle mass using this approach may be in identifying those at risk of progressive sarcopenia and/or frailty, rather than those with already advanced frailty. Barriers to scanning frail participants may persist in other cohorts using an intra-dialytic scanning approach, limiting utility as a screening tool in haemodialysis populations. Scanning either before or after dialysis would potentially mitigate many of these weaknesses. Scanning in a separate office would reduce concerns regarding privacy (many dialysis units utilise temporary curtains only), and in protecting dialysis access and connection to the dialysis machine. However, this would also increase substantially the time spent on the dialysis unit for each participant, impacting the acceptability of investigation to potential participants.

## Conclusions

To conclude, a thigh ultrasound-derived sarcopenia associates with frailty in haemodialysis recipients, but association between frailty and muscle size alone is much more variable. Our study results suggest the utility of quadriceps ultrasound in risk assessment of haemodialysis recipients currently lacks utility in the clinical setting and requires further investigation.

## Supplementary Information

**Additional file 1: Supplementary file 1.** Description of how frailty, vulnerability and robustness defined. **Supplementary Table 1.** Frailty Phenotype. **Supplementary Table 2.** Frailty Index. **Supplementary Table 3.** Edmonton Frailty Scale. **Supplementary Table 4a.** Multiple linear regression of FP by BATT. Model 3. **Supplementary Table 4b.** Multiple linear regression of Frailty Phenotype by Low Muscle Mass. Model 3. **Supplementary Table 4c.** Multiple linear regression of Frailty Phenotype by Sarcopenia. Model 3. **Supplementary Table 5a.** Multiple linear regression of Frailty Index by BATT. Model 3. **Supplementary Table 5b.** Multiple linear regression of Frailty Index by Low Muscle Mass. Model 3. **Supplementary Table 5c.** Multiple linear regression of Frailty Index by Sarcopenia. Model 3. **Supplementary Table 6a.** Multiple linear regression of Edmonton Frailty Scale by BATT. Model 3. **Supplementary Table 6b.** Multiple linear regression of Edmonton Frailty Scale by Low Muscle Mass. Model 3. **Supplementary Table 6c.** Multiple linear regression of Edmonton Frailty Scale by Sarcopenia. Model 3. **Supplementary Table 7a.** Multiple linear regression of Clinical Frailty Scale by BATT. Model 3. **Supplementary Table 7b.** Multiple linear regression of Clinical Frailty Scale by Low Muscle Mass. Model 3. **Supplementary Table 7c.** Multiple linear regression of Clinical Frailty Scale by Sarcopenia. Model 3. **Supplementary Table 8a.** Multivariable logistic regression of Frailty Phenotype by BATT. Model 3. **Supplementary Table 8b.** Multivariable logistic regression of Frailty Phenotype by Low Muscle Mass. Model 3. **Supplementary Table 8c.** Multivariable logistic regression of Frailty Phenotype by Sarcopenia. Model 3. **Supplementary Table 9a.** Multivariable logistic regression of Frailty Index by BATT. Model 3. **Supplementary Table 9b.** Multivariable logistic regression of Frailty Index by Low Muscle Mass. Model 3. **Supplementary Table 9c. **Multivariable logistic regression of Frailty Index by Sarcopenia. Model 3. **Supplementary Table 10a.** Multivariable logistic regression of Edmonton Frailty Scale by BATT. Model 3. **Supplementary Table 10b.** Multivariable logistic regression of Edmonton Frailty Scale by Low Muscle Mass. Model 3. **Supplementary Table 10c.** Multivariable logistic regression of Edmonton Frailty Scale by Sarcopenia. Model 3. **Supplementary Table 11a.** Multivariable logistic regression of Clinical Frailty Scale by BATT. Model 3. **Supplementary Table 11b.** Multivariable logistic regression of Clinical Frailty Scale by Low Muscle Mass. Model 3. **Supplementary Table 11c.** Multivariable logistic regression of Clinical Frailty Scale by Sarcopenia. Model 3. **Supplementary Table 12.** Sensitivity Analyses of Frailty Score by multiple linear regression models in reverse order. **Supplementary Table 13.** Sensitivity analyses of multivariable logistic regression models of frailty. Multivariable models reversed.

## Data Availability

The datasets used and/or analysed during the current study are available from the corresponding author on reasonable request.

## Abbreviations

ADL: Activities of Daily Living
BATT: Bilateral Anterior Thigh Thickness
CFS: Clinical Frailty Scale
CGA: Comprehensive Geriatric Assessment
CT: Computed Tomography
cmRCT: Cohort Multiple Randomised Controlled Trial
DEXA: Dual Energy X-ray Absorptiometry
EFS: Edmonton Frailty Scale
FI: Frailty Index
FP: Frailty Phenotype
GPPAQ: GP Physical Activity Questionnaire
IMD: English Indices of Multiple Deprivation 2015
IQR: Inter-Quartile Range
LMM: Low Muscle Mass
MoCA: Montreal Cognitive Assessment
MRI: Magnetic Resonance Imaging
OR: Odds Ratio
PHQ-9: Patient Health Questionnaire-9
US: Ultrasound
