# Supplementary material for: Ultrasound quadriceps muscle thickness is variably associated with frailty in haemodialysis recipients
Source: BMC Nephrol. 2023 Jan 18;24:16. doi: 10.1186/s12882-022-03043-8 (PMC9847024; doi:10.1186/s12882-022-03043-8)
Supplement: Supplementary file 1 — Additional file 1: Supplementary file 1. Description of how frailty, vulnerability and robustness defined. Supplementary Table 1. Frailty Phenotype. Supplementary Table 2. Frailty Index. Supplementary Table 3. Edmonton Frailty Scale. Supplementary Table 4a. Multiple linear regression of FP by BATT. Model 3. Supplementary Table 4b. Multiple linear regression of Frailty Phenotype by Low Muscle Mass. Model 3. Supplementary Table 4c. Multiple linear regression of Frailty Phenotype by Sarcopenia. Model 3. Supplementary Table 5a. Multiple linear regression of Frailty Index by BATT. Model 3. Supplementary Table 5b. Multiple linear regression of Frailty Index by Low Muscle Mass. Model 3. Supplementary Table 5c. Multiple linear regression of Frailty Index by Sarcopenia. Model 3. Supplementary Table 6a. Multiple linear regression of Edmonton Frailty Scale by BATT. Model 3. Supplementary Table 6b. Multiple linear regression of Edmonton Frailty Scale by Low Muscle Mass. Model 3. Supplementary Table 6c. Multiple linear regression of Edmonton Frailty Scale by Sarcopenia. Model 3. Supplementary Table 7a. Multiple linear regression of Clinical Frailty Scale by BATT. Model 3. Supplementary Table 7b. Multiple linear regression of Clinical Frailty Scale by Low Muscle Mass. Model 3. Supplementary Table 7c. Multiple linear regression of Clinical Frailty Scale by Sarcopenia. Model 3. Supplementary Table 8a. Multivariable logistic regression of Frailty Phenotype by BATT. Model 3. Supplementary Table 8b. Multivariable logistic regression of Frailty Phenotype by Low Muscle Mass. Model 3. Supplementary Table 8c. Multivariable logistic regression of Frailty Phenotype by Sarcopenia. Model 3. Supplementary Table 9a. Multivariable logistic regression of Frailty Index by BATT. Model 3. Supplementary Table 9b. Multivariable logistic regression of Frailty Index by Low Muscle Mass. Model 3. Supplementary Table 9c. Multivariable logistic regression of Frailty Index by Sarcopenia. Model 3. Supplementa [file 12882_2022_3043_MOESM1_ESM.docx]

**Supplementary File 1. Description of how frailty, vulnerability and robustness defined**

### *Frailty Phenotype*

The Frailty Phenotype was determined as a score between zero and five, with participants receiving one point for each of the following: slow walking speed, weak grip strength, exhaustion, weight loss, and low physical activity. Slow walking speed was assessed from a standing start over 4 metres, and participants scored one point if their time was over or equal to prespecified values stratified by height and gender or if they were unable to undertake the test. These values were determined by the original FP described by Fried and colleagues^1^, with times reduced in proportion to the reduced distance of 4m in this study versus 15 feet in the FP study. Poor grip strength was evaluated with dynamometer (Takei Grip D, Takei Scientific Instruments, Niigata, Japan); participants scored one point if their grip strength in either hand was not above cut-off values as per the original FP^1^ stratified by BMI and gender, or if they were unable to perform the test in either hand. Unintentional weight loss of greater than 4.5kg in the past year scored one point. To characterise exhaustion, participants were asked whether they felt that a) everything was an effort or b) they felt they could not get going within the past week. The possible responses were “Rarely”, “Some of the time”, and “Most of the time”. Participants scored one point for exhaustion if they responded that they felt either statement was true either most of the time or - if they answered “Some of the time” – they felt it was the case on more than 3 days in the week prior to recruitment. Physical activity was assessed by response to the question “How often do you engage in activities that require a low or moderate level of energy such as gardening, cleaning the car, or going for a walk?”. Possible responses were “More than once per week”, “Once per week”, “One to three times per month”, and “Hardly ever or never”. Participants scored one point for low physical activity if they responded “One to three times per month” or “Hardly ever or never”. Participants were considered not frail by FP if their total score was 0, vulnerable if their total score was 1-2 and frail if their score was 3-5.

## *Frailty Index*

A Frailty Index score was based upon previous work from van Munster and colleagues conducted in an elderly haemodialysis cohort^2^. The FI consisted of 32 variables across measuring deficits across multiple body systems. Each variable was scored out of one by pre-determined cut-offs and the FI comprised a mean average of these scores, giving a continuous variable between zero and one. A score of $\text{≥}$0.24 was considered frail by FI; a score of ≥0.12 but <0.24 was considered vulnerable and the rest considered robust.

### *Clinical Frailty Scale*

Clinical Frailty Scale (CFS) was derived from interrogation and interpretation of independent activity of daily living questionnaire responses, which were administered by the investigators and interpreted by the lead investigator. Scores can range between 1 to 9, with a threshold score ≥5 considered frail, 4 considered vulnerable and <4 considered robust.

## *Edmonton Frailty Scale*

The Edmonton Frailty Scale was determined according to the original study^3^, but in order to reduce test burden on participants, a 4 metre walk was substituted for the timed up and go test. The results were split into terciles and attributed zero points to those in the lowest tercile, one point to those in the middle tercile and two points to those in the top tercile. To satisfy EFS scoring criteria, the resulting times were split into terciles, with the fastest tercile assigned zero points, the middle tercile one point and the slowest tercile (or unable to walk) two points. A score of ≥8 was considered frail, and a score of 6 or seven vulnerable by EFS.

Supplementary Table 1: Frailty Phenotype

**
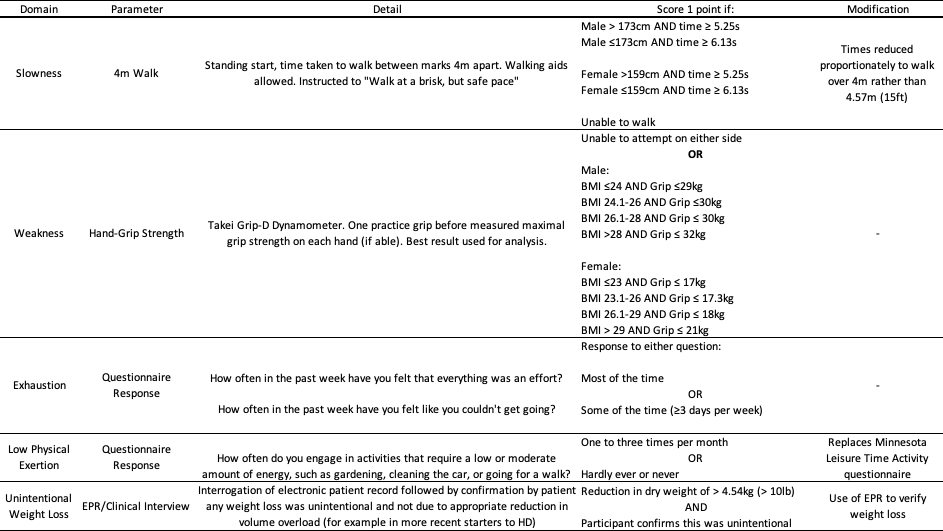
**

Supplementary Table 2: Frailty Index

**
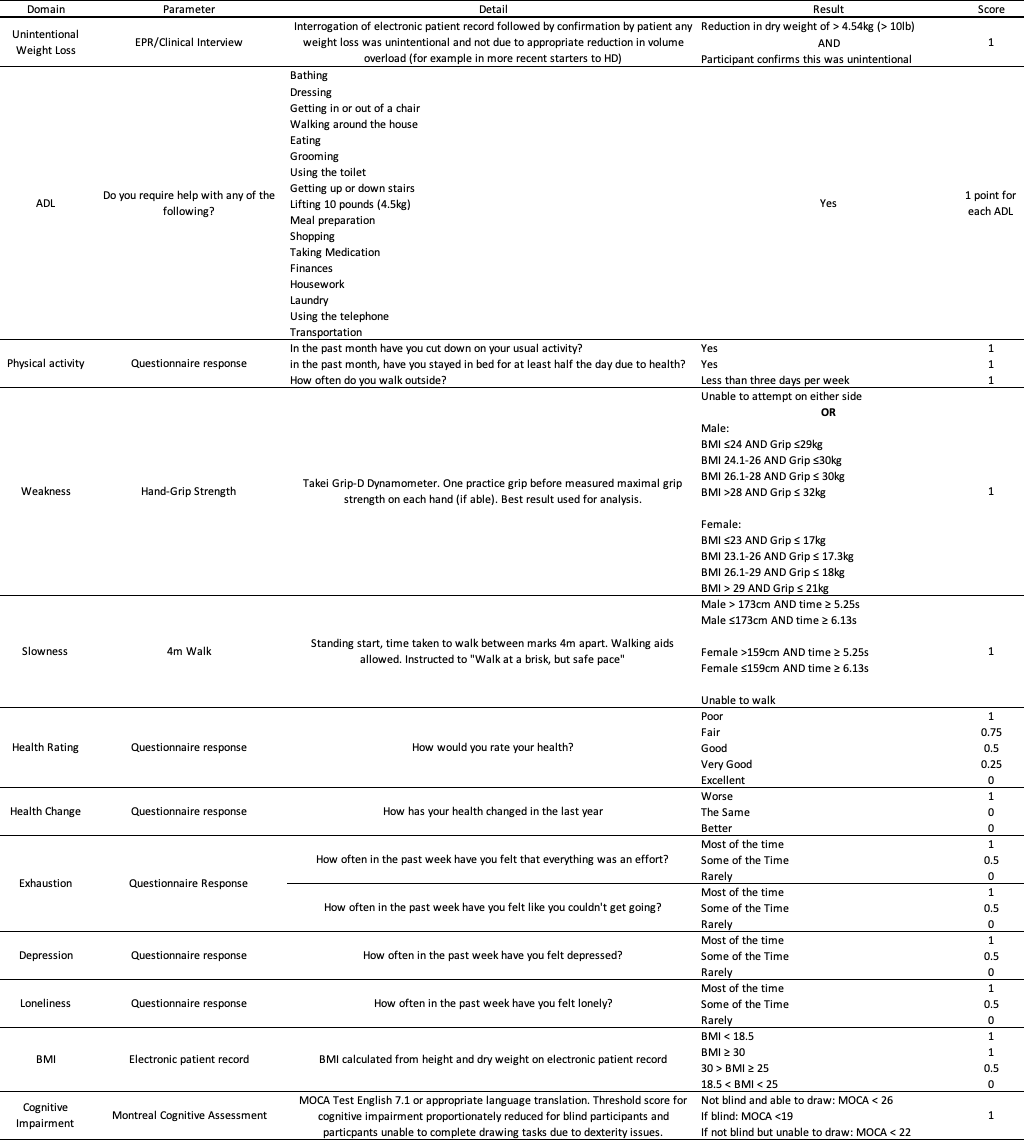
**

Supplementary Table 3: Edmonton Frailty Scale

**
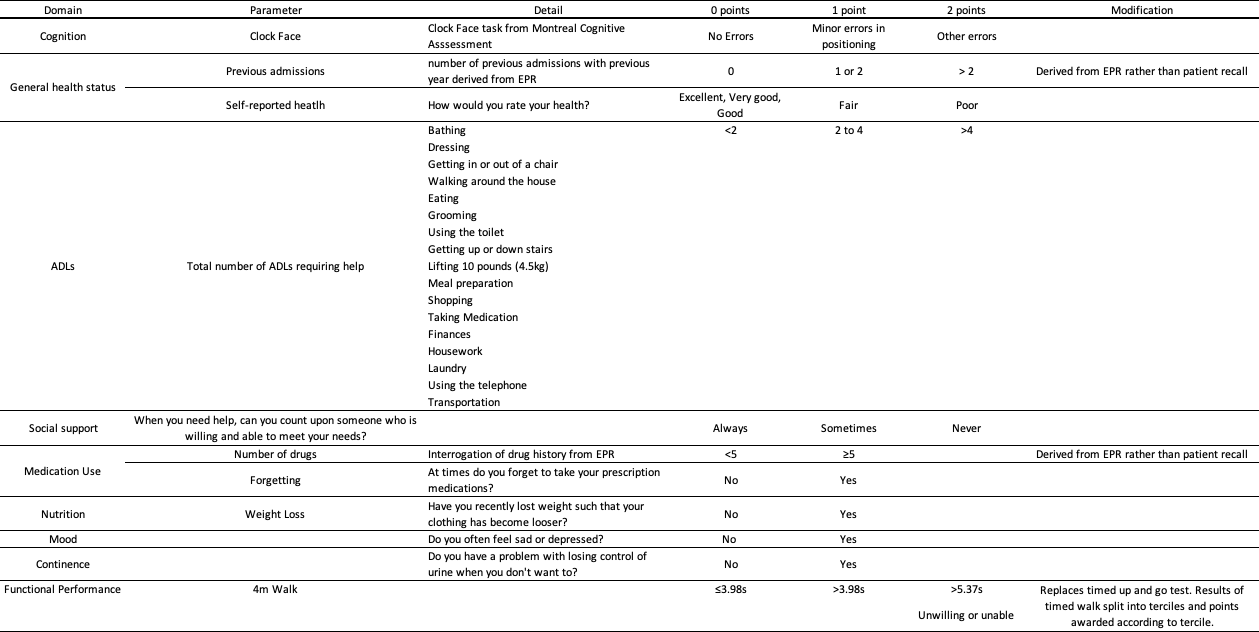
**

Supplementary Table 4a: Multiple linear regression of FP by BATT. Model 3

|  | Coefficient | Lower 95% C.I. | Upper 95% C.I. | P |
| --- | --- | --- | --- | --- |
| BATT | -0.009 | -0.019 | 0.001 | 0.064 |
| Age | 0.001 | -0.013 | 0.015 | 0.935 |
| Ethnicity |  |  |  |  |
| *White* | REFERENCE | | | |
| *South Asian* | **0.406** | **0.014** | **0.798** | **0.043** |
| *Black* | -0.142 | -0.550 | 0.267 | 0.495 |
| *Other* | -0.418 | -1.442 | 0.606 | 0.421 |
| Education Level |  |  |  |  |
| *High School* | REFERENCE | | | |
| *College/6th form* | 0.003 | -0.344 | 0.350 | 0.988 |
| *University* | -0.099 | -0.585 | 0.387 | 0.688 |
| IMD Quintile |  |  |  |  |
| *1* | REFERENCE | | | |
| *2* | 0.164 | -0.251 | 0.580 | 0.436 |
| *3* | -0.014 | -0.416 | 0.388 | 0.945 |
| *4* | -0.293 | -0.808 | 0.222 | 0.263 |
| *5* | -0.425 | -0.900 | 0.050 | 0.079 |
| *Unknown* | 0.047 | -0.501 | 0.596 | 0.865 |
| Gender |  |  |  |  |
| *Male* | REFERENCE | | | |
| *Female* | 0.236 | -0.061 | 0.532 | 0.119 |
| Social Support |  |  |  |  |
| *Yes* | REFERENCE | | | |
| *No* | -0.312 | -0.894 | 0.271 | 0.292 |
| HD Vintage | 0.001 | -0.002 | 0.004 | 0.481 |
| Charlson Index | 0.069 | -0.014 | 0.152 | 0.104 |
| Cognitive Impairment |  |  |  |  |
| *No* | REFERENCE | | | |
| *Yes* | 0.036 | -0.329 | 0.400 | 0.847 |
| PHQ-9 Score | **0.061** | **0.031** | **0.091** | **<0.001** |
| Self-Reported Health Change |  |  |  |  |
| *Better* | REFERENCE | | | |
| *The Same* | -0.093 | -0.489 | 0.303 | 0.643 |
| *Worse* | -0.095 | -0.483 | 0.293 | 0.630 |
| Self-Reported Health Today | **-0.011** | **-0.018** | **-0.003** | **0.006** |
| Walking Aid Use |  |  |  |  |
| *No* | REFERENCE | | | |
| *Yes* | **0.848** | **0.532** | **1.163** | **<0.001** |
| Self-Reported Slow Walk Speed |  |  |  |  |
| *No* | REFERENCE | | | |
| *Yes* | **0.625** | **0.305** | **0.945** | **<0.001** |
| Constant | **1.548** | **0.377** | **2.719** | **0.010** |

Supplementary Table 4b: Multiple linear regression of Frailty Phenotype by Low Muscle Mass. Model 3

|  | Coefficient | Lower 95% C.I. | Upper 95% C.I. | P |
| --- | --- | --- | --- | --- |
| Low Muscle Mass |  |  |  |  |
| *No* | REFERENCE | | | |
| *Yes* | **0.379** | **0.082** | **0.676** | **0.013** |
| Age | 0.002 | -0.012 | 0.015 | 0.829 |
| Ethnicity |  |  |  |  |
| *White* | REFERENCE | | | |
| *South Asian* | **0.418** | **0.030** | **0.806** | **0.035** |
| *Black* | -0.116 | -0.523 | 0.290 | 0.573 |
| *Other* | -0.424 | -1.439 | 0.591 | 0.411 |
| Education Level |  |  |  |  |
| *High School* | REFERENCE | | | |
| *College/6th form* | 0.040 | -0.307 | 0.387 | 0.821 |
| *University* | -0.073 | -0.557 | 0.410 | 0.765 |
| IMD Quintile |  |  |  |  |
| *1* | REFERENCE | | | |
| *2* | 0.095 | -0.321 | 0.510 | 0.653 |
| *3* | -0.041 | -0.441 | 0.360 | 0.841 |
| *4* | -0.278 | -0.789 | 0.233 | 0.284 |
| *5* | **-0.486** | **-0.958** | **-0.014** | **0.044** |
| *Unknown* | 0.042 | -0.502 | 0.587 | 0.879 |
| Gender |  |  |  |  |
| *Male* | REFERENCE | | | |
| *Female* | **0.392** | **0.098** | **0.686** | **0.009** |
| Social Support |  |  |  |  |
| *Yes* | REFERENCE | | | |
| *No* | -0.309 | -0.886 | 0.269 | 0.293 |
| HD Vintage | 0.001 | -0.002 | 0.003 | 0.634 |
| Charlson Index | 0.070 | -0.012 | 0.152 | 0.094 |
| Cognitive Impairment |  |  |  |  |
| *No* | REFERENCE | | | |
| *Yes* | 0.035 | -0.325 | 0.396 | 0.847 |
| PHQ-9 Score | **0.062** | **0.032** | **0.092** | **<0.001** |
| Self-Reported Health Change |  |  |  |  |
| *Better* | REFERENCE | | | |
| *The Same* | -0.117 | -0.510 | 0.277 | 0.559 |
| *Worse* | -0.078 | -0.463 | 0.307 | 0.690 |
| Self-Reported Health Today | **-0.010** | **-0.017** | **-0.002** | **0.012** |
| Walking Aid Use |  |  |  |  |
| *No* | REFERENCE | | | |
| *Yes* | **0.814** | **0.499** | **1.130** | **<0.001** |
| Self-Reported Slow Walk Speed |  |  |  |  |
| *No* | REFERENCE | | | |
| *Yes* | **0.657** | **0.341** | **0.974** | **<0.001** |
| Constant | 0.717 | -0.322 | 1.756 | 0.175 |

Supplementary Table 4c: Multiple linear regression of Frailty Phenotype by Sarcopenia. Model 3

|  | Coefficient | Lower 95% C.I. | Upper 95% C.I. | P |
| --- | --- | --- | --- | --- |
| Sarcopenia |  |  |  |  |
| *No* | REFERENCE | | | |
| *Yes* | 0.022 | -0.017 | 0.061 | 0.265 |
| Age | -0.001 | -0.003 | 0.000 | 0.086 |
| Ethnicity |  |  |  |  |
| *White* | REFERENCE | | | |
| *South Asian* | **0.057** | **0.010** | **0.105** | **0.019** |
| *Black* | 0.014 | -0.036 | 0.064 | 0.576 |
| *Other* | 0.000 | -0.125 | 0.124 | 0.994 |
| Education Level |  |  |  |  |
| *High School* | REFERENCE | | | |
| *College/6th form* | -0.001 | -0.043 | 0.041 | 0.962 |
| *University* | -0.009 | -0.068 | 0.050 | 0.771 |
| IMD Quintile |  |  |  |  |
| *1* | REFERENCE | | | |
| *2* | 0.016 | -0.035 | 0.066 | 0.539 |
| *3* | 0.020 | -0.029 | 0.069 | 0.430 |
| *4* | -0.003 | -0.066 | 0.060 | 0.918 |
| *5* | -0.008 | -0.066 | 0.050 | 0.776 |
| *Unknown* | 0.011 | -0.056 | 0.078 | 0.739 |
| Gender |  |  |  |  |
| *Male* | REFERENCE | | | |
| *Female* | **0.045** | **0.010** | **0.080** | **0.012** |
| Social Support |  |  |  |  |
| *Yes* | REFERENCE | | | |
| *No* | **-0.106** | **-0.177** | **-0.035** | **0.004** |
| HD Vintage | 0.000 | 0.000 | 0.000 | 0.820 |
| Charlson Index | **0.016** | **0.006** | **0.026** | **0.002** |
| Cognitive Impairment |  |  |  |  |
| *No* | REFERENCE | | | |
| *Yes* | **0.063** | **0.019** | **0.107** | **0.006** |
| PHQ-9 Score | **0.012** | **0.009** | **0.016** | **<0.001** |
| Self-Reported Health Change |  |  |  |  |
| *Better* | REFERENCE | | | |
| *The Same* | **-0.051** | **-0.100** | **-0.003** | **0.038** |
| *Worse* | 0.022 | -0.025 | 0.069 | 0.364 |
| Self-Reported Health Today | -0.001 | -0.002 | 0.000 | 0.204 |
| Walking Aid Use |  |  |  |  |
| *No* | REFERENCE | | | |
| *Yes* | **0.121** | **0.082** | **0.159** | **<0.001** |
| Self-Reported Slow Walk Speed |  |  |  |  |
| *No* | REFERENCE | | | |
| *Yes* | **0.068** | **0.029** | **0.107** | **0.001** |
| Constant | 0.116 | -0.009 | 0.241 | 0.069 |

Supplementary Table 5a: Multiple linear regression of Frailty Index by BATT. Model 3

|  | Coefficient | Lower 95% C.I. | Upper 95% C.I. | P |
| --- | --- | --- | --- | --- |
| BATT | 0.001 | -0.001 | 0.002 | 0.355 |
| Age | -0.001 | -0.003 | 0.000 | 0.109 |
| Ethnicity |  |  |  |  |
| *White* | REFERENCE | | | |
| *South Asian* | **0.061** | **0.013** | **0.109** | **0.012** |
| *Black* | 0.010 | -0.039 | 0.060 | 0.682 |
| *Other* | 0.009 | -0.116 | 0.134 | 0.889 |
| Education Level |  |  |  |  |
| *High School* | REFERENCE | | | |
| *College/6th form* | -0.005 | -0.048 | 0.037 | 0.806 |
| *University* | -0.012 | -0.072 | 0.047 | 0.679 |
| IMD Quintile |  |  |  |  |
| *1* | REFERENCE | | | |
| *2* | 0.016 | -0.035 | 0.067 | 0.536 |
| *3* | 0.019 | -0.030 | 0.068 | 0.438 |
| *4* | -0.005 | -0.068 | 0.058 | 0.881 |
| *5* | -0.012 | -0.070 | 0.046 | 0.691 |
| *Unknown* | 0.012 | -0.055 | 0.079 | 0.728 |
| Gender |  |  |  |  |
| *Male* | REFERENCE | | | |
| *Female* | **0.048** | **0.012** | **0.084** | **0.010** |
| Social Support |  |  |  |  |
| *Yes* | REFERENCE | | | |
| *No* | **-0.103** | **-0.174** | **-0.032** | **0.005** |
| HD Vintage | 0.000 | 0.000 | 0.000 | 0.556 |
| Charlson Index | **0.017** | **0.007** | **0.027** | **0.001** |
| Cognitive Impairment |  |  |  |  |
| *No* | REFERENCE | | | |
| *Yes* | **0.060** | **0.015** | **0.104** | **0.009** |
| PHQ-9 Score | **0.012** | **0.009** | **0.016** | **<0.001** |
| Self-Reported Health Change |  |  |  |  |
| *Better* | REFERENCE | | | |
| *The Same* | -0.048 | -0.096 | 0.000 | 0.051 |
| *Worse* | 0.023 | -0.025 | 0.070 | 0.343 |
| Self-Reported Health Today | -0.001 | -0.002 | 0.000 | 0.100 |
| Walking Aid Use |  |  |  |  |
| *No* | REFERENCE | | | |
| *Yes* | **0.124** | **0.085** | **0.162** | **<0.001** |
| Self-Reported Slow Walk Speed |  |  |  |  |
| *No* | REFERENCE | | | |
| *Yes* | **0.069** | **0.030** | **0.108** | **0.001** |
| Constant | 0.090 | -0.052 | 0.233 | 0.213 |

Supplementary Table 5b: Multiple linear regression of Frailty Index by Low Muscle Mass. Model 3

|  | Coefficient | Lower 95% C.I. | Upper 95% C.I. | P |
| --- | --- | --- | --- | --- |
| Low Muscle Mass |  |  |  |  |
| *No* | REFERENCE | | | |
| *Yes* | -0.022 | -0.059 | 0.014 | 0.230 |
| Age | -0.001 | -0.003 | 0.000 | 0.094 |
| Ethnicity |  |  |  |  |
| *White* | REFERENCE | | | |
| *South Asian* | **0.060** | **0.013** | **0.108** | **0.013** |
| *Black* | 0.009 | -0.041 | 0.059 | 0.724 |
| *Other* | 0.009 | -0.116 | 0.134 | 0.886 |
| Education Level |  |  |  |  |
| *High School* | REFERENCE | | | |
| *College/6th form* | -0.007 | -0.050 | 0.035 | 0.734 |
| *University* | -0.014 | -0.073 | 0.045 | 0.645 |
| IMD Quintile |  |  |  |  |
| *1* | REFERENCE | | | |
| *2* | 0.020 | -0.031 | 0.071 | 0.440 |
| *3* | 0.021 | -0.028 | 0.070 | 0.405 |
| *4* | -0.006 | -0.068 | 0.057 | 0.858 |
| *5* | -0.008 | -0.066 | 0.050 | 0.783 |
| *Unknown* | 0.012 | -0.055 | 0.079 | 0.721 |
| Gender |  |  |  |  |
| *Male* | REFERENCE | | | |
| *Female* | **0.039** | **0.002** | **0.075** | **0.036** |
| Social Support |  |  |  |  |
| *Yes* | REFERENCE | | | |
| *No* | **-0.103** | **-0.174** | **-0.032** | **0.005** |
| HD Vintage | 0.000 | 0.000 | 0.000 | 0.492 |
| Charlson Index | **0.017** | **0.007** | **0.027** | **0.001** |
| Cognitive Impairment |  |  |  |  |
| *No* | REFERENCE | | | |
| *Yes* | **0.060** | **0.016** | **0.104** | **0.008** |
| PHQ-9 Score | **0.012** | **0.008** | **0.016** | **<0.001** |
| Self-Reported Health Change |  |  |  |  |
| *Better* | REFERENCE | | | |
| *The Same* | -0.047 | -0.095 | 0.002 | 0.058 |
| *Worse* | 0.022 | -0.025 | 0.069 | 0.364 |
| Self-Reported Health Today | -0.001 | -0.002 | 0.000 | 0.080 |
| Walking Aid Use |  |  |  |  |
| *No* | REFERENCE | | | |
| *Yes* | **0.126** | **0.087** | **0.164** | **<0.001** |
| Self-Reported Slow Walk Speed |  |  |  |  |
| *No* | REFERENCE | | | |
| *Yes* | **0.067** | **0.028** | **0.106** | **0.001** |
| Constant | **0.140** | **0.013** | **0.268** | **0.031** |

Supplementary Table 5c: Multiple linear regression of Frailty Index by Sarcopenia. Model 3

|  | Coefficient | Lower 95% C.I. | Upper 95% C.I. | P |
| --- | --- | --- | --- | --- |
| Sarcopenia |  |  |  |  |
| *No* | REFERENCE | | | |
| *Yes* | 0.022 | -0.017 | 0.061 | 0.265 |
| Age | -0.001 | -0.003 | 0.000 | 0.086 |
| Ethnicity |  |  |  |  |
| *White* | REFERENCE | | | |
| *South Asian* | **0.057** | **0.010** | **0.105** | **0.019** |
| *Black* | 0.014 | -0.036 | 0.064 | 0.576 |
| *Other* | 0.000 | -0.125 | 0.124 | 0.994 |
| Education Level |  |  |  |  |
| *High School* | REFERENCE | | | |
| *College/6th form* | -0.001 | -0.043 | 0.041 | 0.962 |
| *University* | -0.009 | -0.068 | 0.050 | 0.771 |
| IMD Quintile |  |  |  |  |
| *1* | REFERENCE | | | |
| *2* | 0.016 | -0.035 | 0.066 | 0.539 |
| *3* | 0.020 | -0.029 | 0.069 | 0.430 |
| *4* | -0.003 | -0.066 | 0.060 | 0.918 |
| *5* | -0.008 | -0.066 | 0.050 | 0.776 |
| *Unknown* | 0.011 | -0.056 | 0.078 | 0.739 |
| Gender |  |  |  |  |
| *Male* | REFERENCE | | | |
| *Female* | **0.045** | **0.010** | **0.080** | **0.012** |
| Social Support |  |  |  |  |
| *Yes* | REFERENCE | | | |
| *No* | **-0.106** | **-0.177** | **-0.035** | **0.004** |
| HD Vintage | 0.000 | 0.000 | 0.000 | 0.820 |
| Charlson Index | **0.016** | **0.006** | **0.026** | **0.002** |
| Cognitive Impairment |  |  |  |  |
| *No* | REFERENCE | | | |
| *Yes* | **0.063** | **0.019** | **0.107** | **0.006** |
| PHQ-9 Score | **0.012** | **0.009** | **0.016** | **<0.001** |
| Self-Reported Health Change |  |  |  |  |
| *Better* | REFERENCE | | | |
| *The Same* | **-0.051** | **-0.100** | **-0.003** | **0.038** |
| *Worse* | 0.022 | -0.025 | 0.069 | 0.364 |
| Self-Reported Health Today | -0.001 | -0.002 | 0.000 | 0.204 |
| Walking Aid Use |  |  |  |  |
| *No* | REFERENCE | | | |
| *Yes* | **0.121** | **0.082** | **0.159** | **<0.001** |
| Self-Reported Slow Walk Speed |  |  |  |  |
| *No* | REFERENCE | | | |
| *Yes* | **0.068** | **0.029** | **0.107** | **0.001** |
| Constant | 0.116 | -0.009 | 0.241 | 0.069 |

Supplementary Table 6a: Multiple linear regression of Edmonton Frailty Scale by BATT. Model 3

|  | Coefficient | Lower 95% C.I. | Upper 95% C.I. | P |
| --- | --- | --- | --- | --- |
| BATT | -0.020 | -0.042 | 0.001 | 0.064 |
| Age | -0.017 | -0.047 | 0.014 | 0.288 |
| Ethnicity |  |  |  |  |
| *White* | REFERENCE | | | |
| *South Asian* | 0.477 | -0.382 | 1.337 | 0.274 |
| *Black* | **0.908** | **0.012** | **1.804** | **0.047** |
| *Other* | -1.760 | -4.005 | 0.484 | 0.123 |
| Education Level |  |  |  |  |
| *High School* | REFERENCE | | | |
| *College/6th form* | -0.290 | -1.050 | 0.471 | 0.453 |
| *University* | -0.539 | -1.605 | 0.527 | 0.320 |
| IMD Quintile |  |  |  |  |
| *1* | REFERENCE | | | |
| *2* | 0.651 | -0.259 | 1.561 | 0.160 |
| *3* | -0.403 | -1.285 | 0.478 | 0.368 |
| *4* | -0.187 | -1.316 | 0.942 | 0.744 |
| *5* | 0.431 | -0.610 | 1.472 | 0.415 |
| *Unknown* | -0.492 | -1.695 | 0.711 | 0.421 |
| Gender |  |  |  |  |
| *Male* | REFERENCE | | | |
| *Female* | 0.187 | -0.463 | 0.838 | 0.570 |
| Social Support |  |  |  |  |
| *Yes* | REFERENCE | | | |
| *No* | 0.958 | -0.318 | 2.234 | 0.140 |
| HD Vintage | -0.004 | -0.010 | 0.002 | 0.150 |
| Charlson Index | 0.182 | 0.000 | 0.363 | 0.050 |
| Cognitive Impairment |  |  |  |  |
| *No* | REFERENCE | | | |
| *Yes* | 0.681 | -0.118 | 1.481 | 0.094 |
| PHQ-9 Score | **0.130** | **0.064** | **0.196** | **<0.001** |
| Self-Reported Health Change |  |  |  |  |
| *Better* | REFERENCE | | | |
| *The Same* | **-1.289** | **-2.156** | **-0.421** | **0.004** |
| *Worse* | 0.209 | -0.642 | 1.060 | 0.628 |
| Self-Reported Health Today | -0.014 | -0.030 | 0.003 | 0.099 |
| Walking Aid Use |  |  |  |  |
| *No* | REFERENCE | | | |
| *Yes* | **1.551** | **0.860** | **2.242** | **<0.001** |
| Self-Reported Slow Walk Speed |  |  |  |  |
| *No* | REFERENCE | | | |
| *Yes* | **0.824** | **0.123** | **1.525** | **0.021** |
| Constant | **7.429** | **4.863** | **9.995** | **<0.001** |

Supplementary Table 6b: Multiple linear regression of Edmonton Frailty Scale by Low Muscle Mass. Model 3

|  | Coefficient | Lower 95% C.I. | Upper 95% C.I. | P |
| --- | --- | --- | --- | --- |
| Low Muscle Mass |  |  |  |  |
| *No* | REFERENCE | | | |
| *Yes* | 0.277 | -0.383 | 0.938 | 0.408 |
| Age | -0.015 | -0.046 | 0.016 | 0.350 |
| Ethnicity |  |  |  |  |
| *White* | REFERENCE | | | |
| *South Asian* | 0.524 | -0.340 | 1.388 | 0.233 |
| *Black* | 0.901 | -0.004 | 1.806 | 0.051 |
| *Other* | -1.659 | -3.919 | 0.600 | 0.149 |
| Education Level |  |  |  |  |
| *High School* | REFERENCE | | | |
| *College/6th form* | -0.329 | -1.101 | 0.444 | 0.402 |
| *University* | -0.563 | -1.639 | 0.513 | 0.304 |
| IMD Quintile |  |  |  |  |
| *1* | REFERENCE | | | |
| *2* | 0.596 | -0.328 | 1.521 | 0.205 |
| *3* | -0.365 | -1.256 | 0.526 | 0.420 |
| *4* | -0.154 | -1.291 | 0.983 | 0.790 |
| *5* | 0.359 | -0.691 | 1.409 | 0.501 |
| *Unknown* | -0.494 | -1.706 | 0.718 | 0.422 |
| Gender |  |  |  |  |
| *Male* | REFERENCE | | | |
| *Female* | 0.400 | -0.254 | 1.055 | 0.229 |
| Social Support |  |  |  |  |
| *Yes* | REFERENCE | | | |
| *No* | 0.955 | -0.331 | 2.241 | 0.145 |
| HD Vintage | -0.004 | -0.010 | 0.002 | 0.194 |
| Charlson Index | **0.198** | **0.016** | **0.380** | **0.033** |
| Cognitive Impairment |  |  |  |  |
| *No* | REFERENCE | | | |
| *Yes* | 0.621 | -0.182 | 1.424 | 0.129 |
| PHQ-9 Score | **0.126** | **0.059** | **0.192** | **<0.001** |
| Self-Reported Health Change |  |  |  |  |
| *Better* | REFERENCE | | | |
| *The Same* | **-1.295** | **-2.171** | **-0.420** | **0.004** |
| *Worse* | 0.248 | -0.609 | 1.105 | 0.568 |
| Self-Reported Health Today | -0.014 | -0.030 | 0.003 | 0.099 |
| Walking Aid Use |  |  |  |  |
| *No* | REFERENCE | | | |
| *Yes* | **1.575** | **0.873** | **2.278** | **<0.001** |
| Self-Reported Slow Walk Speed |  |  |  |  |
| *No* | REFERENCE | | | |
| *Yes* | **0.876** | **0.171** | **1.581** | **0.015** |
| Constant | **6.034** | **3.721** | **8.347** | **<0.001** |

Supplementary Table 6c: Multiple linear regression of Edmonton Frailty Scale by Sarcopenia. Model 3

|  | Coefficient | Lower 95% C.I. | Upper 95% C.I. | P |
| --- | --- | --- | --- | --- |
| Sarcopenia |  |  |  |  |
| *No* | REFERENCE | | | |
| *Yes* | **1.101** | **0.411** | **1.791** | **0.002** |
| Age | -0.017 | -0.047 | 0.013 | 0.268 |
| Ethnicity |  |  |  |  |
| *White* | REFERENCE | | | |
| *South Asian* | 0.425 | -0.420 | 1.270 | 0.323 |
| *Black* | **1.008** | **0.124** | **1.892** | **0.026** |
| *Other* | -1.848 | -4.053 | 0.357 | 0.100 |
| Education Level |  |  |  |  |
| *High School* | REFERENCE | | | |
| *College/6th form* | -0.314 | -1.056 | 0.427 | 0.404 |
| *University* | -0.507 | -1.554 | 0.540 | 0.340 |
| IMD Quintile |  |  |  |  |
| *1* | REFERENCE | | | |
| *2* | 0.631 | -0.264 | 1.526 | 0.166 |
| *3* | -0.180 | -1.046 | 0.686 | 0.682 |
| *4* | -0.032 | -1.144 | 1.080 | 0.955 |
| *5* | 0.498 | -0.526 | 1.523 | 0.339 |
| *Unknown* | -0.511 | -1.693 | 0.671 | 0.395 |
| Gender |  |  |  |  |
| *Male* | REFERENCE | | | |
| *Female* | 0.402 | -0.220 | 1.024 | 0.204 |
| Social Support |  |  |  |  |
| *Yes* | REFERENCE | | | |
| *No* | 0.783 | -0.476 | 2.042 | 0.221 |
| HD Vintage | -0.005 | -0.011 | 0.001 | 0.079 |
| Charlson Index | 0.170 | -0.008 | 0.349 | 0.061 |
| Cognitive Impairment |  |  |  |  |
| *No* | REFERENCE | | | |
| *Yes* | 0.610 | -0.170 | 1.390 | 0.125 |
| PHQ-9 Score | **0.125** | **0.060** | **0.189** | **<0.001** |
| Self-Reported Health Change |  |  |  |  |
| *Better* | REFERENCE | | | |
| *The Same* | **-1.409** | **-2.266** | **-0.552** | **0.001** |
| *Worse* | 0.251 | -0.584 | 1.087 | 0.554 |
| Self-Reported Health Today | -0.009 | -0.025 | 0.007 | 0.273 |
| Walking Aid Use |  |  |  |  |
| *No* | REFERENCE | | | |
| *Yes* | **1.577** | **0.901** | **2.253** | **<0.001** |
| Self-Reported Slow Walk Speed |  |  |  |  |
| *No* | REFERENCE | | | |
| *Yes* | **0.895** | **0.207** | **1.583** | **0.011** |
| Constant | **5.889** | **3.677** | **8.101** | **<0.001** |

Supplementary Table 7a: Multiple linear regression of Clinical Frailty Scale by BATT. Model 3

|  | Coefficient | Lower 95% C.I. | Upper 95% C.I. | P |
| --- | --- | --- | --- | --- |
| BATT | 0.001 | -0.011 | 0.012 | 0.912 |
| Age | 0.001 | -0.015 | 0.018 | 0.858 |
| Ethnicity |  |  |  |  |
| *White* | REFERENCE | | | |
| *South Asian* | **0.549** | **0.099** | **0.998** | **0.017** |
| *Black* | -0.070 | -0.539 | 0.398 | 0.768 |
| *Other* | 0.378 | -0.796 | 1.552 | 0.526 |
| Education Level |  |  |  |  |
| *High School* | REFERENCE | | | |
| *College/6th form* | 0.126 | -0.271 | 0.524 | 0.531 |
| *University* | -0.123 | -0.680 | 0.435 | 0.665 |
| IMD Quintile |  |  |  |  |
| *1* | REFERENCE | | | |
| *2* | 0.065 | -0.411 | 0.541 | 0.788 |
| *3* | 0.226 | -0.235 | 0.687 | 0.334 |
| *4* | -0.472 | -1.062 | 0.119 | 0.117 |
| *5* | -0.309 | -0.853 | 0.235 | 0.264 |
| *Unknown* | 0.102 | -0.527 | 0.731 | 0.749 |
| Gender |  |  |  |  |
| *Male* | REFERENCE | | | |
| *Female* | **0.368** | **0.028** | **0.708** | **0.034** |
| Social Support |  |  |  |  |
| *Yes* | REFERENCE | | | |
| *No* | -0.727 | -1.394 | -0.059 | 0.033 |
| HD Vintage | 0.000 | -0.003 | 0.003 | 0.977 |
| Charlson Index | **0.097** | **0.002** | **0.192** | **0.045** |
| Cognitive Impairment |  |  |  |  |
| *No* | REFERENCE | | | |
| *Yes* | 0.391 | -0.027 | 0.809 | 0.066 |
| PHQ-9 Score | **0.057** | **0.022** | **0.091** | **0.001** |
| Self-Reported Health Change |  |  |  |  |
| *Better* | REFERENCE | | | |
| *The Same* | -0.250 | -0.703 | 0.204 | 0.279 |
| *Worse* | -0.042 | -0.487 | 0.403 | 0.853 |
| Self-Reported Health Today | -0.006 | -0.014 | 0.003 | 0.166 |
| Walking Aid Use |  |  |  |  |
| *No* | REFERENCE | | | |
| *Yes* | **0.828** | **0.467** | **1.190** | **<0.001** |
| Self-Reported Slow Walk Speed |  |  |  |  |
| *No* | REFERENCE | | | |
| *Yes* | **0.417** | **0.050** | **0.783** | **0.026** |
| Constant | **2.776** | **1.434** | **4.118** | **<0.001** |

Supplementary Table 7b: Multiple linear regression of Clinical Frailty Scale by Low Muscle Mass. Model 3

|  | Coefficient | Lower 95% C.I. | Upper 95% C.I. | P |
| --- | --- | --- | --- | --- |
| Low Muscle Mass |  |  |  |  |
| *No* | REFERENCE | | | |
| *Yes* | -0.074 | -0.416 | 0.269 | 0.672 |
| Age | 0.001 | -0.015 | 0.017 | 0.865 |
| Ethnicity |  |  |  |  |
| *White* | REFERENCE | | | |
| *South Asian* | **0.550** | **0.101** | **0.998** | **0.017** |
| *Black* | -0.077 | -0.547 | 0.392 | 0.746 |
| *Other* | 0.388 | -0.784 | 1.560 | 0.514 |
| Education Level |  |  |  |  |
| *High School* | REFERENCE | | | |
| *College/6th form* | 0.113 | -0.287 | 0.514 | 0.577 |
| *University* | -0.131 | -0.689 | 0.427 | 0.643 |
| IMD Quintile |  |  |  |  |
| *1* | REFERENCE | | | |
| *2* | 0.078 | -0.402 | 0.558 | 0.748 |
| *3* | 0.236 | -0.226 | 0.699 | 0.314 |
| *4* | -0.473 | -1.062 | 0.117 | 0.116 |
| *5* | -0.300 | -0.845 | 0.245 | 0.279 |
| *Unknown* | 0.103 | -0.525 | 0.732 | 0.746 |
| Gender |  |  |  |  |
| *Male* | REFERENCE | | | |
| *Female* | **0.346** | **0.007** | **0.686** | **0.046** |
| Social Support |  |  |  |  |
| *Yes* | REFERENCE | | | |
| *No* | -0.728 | -1.395 | -0.061 | 0.033 |
| HD Vintage | 0.000 | -0.003 | 0.003 | 0.921 |
| Charlson Index | **0.098** | **0.004** | **0.193** | **0.042** |
| Cognitive Impairment |  |  |  |  |
| *No* | REFERENCE | | | |
| *Yes* | 0.386 | -0.030 | 0.802 | 0.069 |
| PHQ-9 Score | **0.056** | **0.021** | **0.090** | **0.002** |
| Self-Reported Health Change |  |  |  |  |
| *Better* | REFERENCE | | | |
| *The Same* | -0.244 | -0.698 | 0.210 | 0.290 |
| *Worse* | -0.043 | -0.487 | 0.401 | 0.849 |
| Self-Reported Health Today | -0.006 | -0.015 | 0.002 | 0.152 |
| Walking Aid Use |  |  |  |  |
| *No* | REFERENCE | | | |
| *Yes* | **0.839** | **0.475** | **1.203** | **<0.001** |
| Self-Reported Slow Walk Speed |  |  |  |  |
| *No* | REFERENCE | | | |
| *Yes* | **0.413** | **0.047** | **0.779** | **0.027** |
| Constant | **2.869** | **1.670** | **4.069** | **<0.001** |

Supplementary Table 7c: Multiple linear regression of Clinical Frailty Scale by Sarcopenia. Model 3

|  | Coefficient | Lower 95% C.I. | Upper 95% C.I. | P |
| --- | --- | --- | --- | --- |
| Sarcopenia |  |  |  |  |
| *No* | REFERENCE | | | |
| *Yes* | 0.104 | -0.263 | 0.471 | 0.575 |
| Age | 0.001 | -0.015 | 0.017 | 0.883 |
| Ethnicity |  |  |  |  |
| *White* | REFERENCE | | | |
| *South Asian* | **0.536** | **0.087** | **0.986** | **0.020** |
| *Black* | -0.056 | -0.526 | 0.414 | 0.814 |
| *Other* | 0.350 | -0.823 | 1.522 | 0.557 |
| Education Level |  |  |  |  |
| *High School* | REFERENCE | | | |
| *College/6th form* | 0.136 | -0.258 | 0.531 | 0.495 |
| *University* | -0.111 | -0.668 | 0.445 | 0.693 |
| IMD Quintile |  |  |  |  |
| *1* | REFERENCE | | | |
| *2* | 0.064 | -0.412 | 0.539 | 0.792 |
| *3* | 0.236 | -0.224 | 0.697 | 0.312 |
| *4* | -0.461 | -1.052 | 0.130 | 0.125 |
| *5* | -0.298 | -0.842 | 0.247 | 0.282 |
| *Unknown* | 0.100 | -0.528 | 0.729 | 0.753 |
| Gender |  |  |  |  |
| *Male* | REFERENCE | | | |
| *Female* | **0.370** | **0.039** | **0.700** | **0.029** |
| Social Support |  |  |  |  |
| *Yes* | REFERENCE | | | |
| *No* | **-0.742** | **-1.411** | **-0.073** | **0.030** |
| HD Vintage | 0.000 | -0.003 | 0.003 | 0.924 |
| Charlson Index | 0.093 | -0.002 | 0.188 | 0.054 |
| Cognitive Impairment |  |  |  |  |
| *No* | REFERENCE | | | |
| *Yes* | 0.396 | -0.019 | 0.810 | 0.061 |
| PHQ-9 Score | **0.057** | **0.023** | **0.091** | **0.001** |
| Self-Reported Health Change |  |  |  |  |
| *Better* | REFERENCE | | | |
| *The Same* | -0.263 | -0.718 | 0.192 | 0.256 |
| *Worse* | -0.043 | -0.487 | 0.401 | 0.849 |
| Self-Reported Health Today | -0.005 | -0.014 | 0.003 | 0.224 |
| Walking Aid Use |  |  |  |  |
| *No* | REFERENCE | | | |
| *Yes* | **0.822** | **0.462** | **1.181** | **<0.001** |
| Self-Reported Slow Walk Speed |  |  |  |  |
| *No* | REFERENCE | | | |
| *Yes* | **0.418** | **0.053** | **0.784** | **0.025** |
| Constant | **2.779** | **1.603** | **3.955** | **<0.001** |

Supplementary Table 8a: Multivariable logistic regression of Frailty Phenotype by BATT. Model 3

|  | OR | Lower 95% C.I. | Upper 95% C.I. | P |
| --- | --- | --- | --- | --- |
| BATT | 0.965 | 0.930 | 1.001 | 0.060 |
| Age | 1.002 | 0.953 | 1.054 | 0.938 |
| Ethnicity |  |  |  |  |
| *White* | REFERENCE | | | |
| *South Asian* | 1.383 | 0.353 | 5.409 | 0.642 |
| *Black* | 0.456 | 0.111 | 1.866 | 0.275 |
| *Other* | OMITTED | | | |
| Education Level |  |  |  |  |
| *High School* | REFERENCE | | | |
| *College/6th form* | 0.508 | 0.171 | 1.510 | 0.223 |
| *University* | 0.087 | 0.006 | 1.265 | 0.074 |
| IMD Quintile |  |  |  |  |
| *1* | REFERENCE | | | |
| *2* | 1.898 | 0.476 | 7.569 | 0.364 |
| *3* | 0.661 | 0.184 | 2.374 | 0.526 |
| *4* | 0.170 | 0.024 | 1.231 | 0.079 |
| *5* | 0.204 | 0.037 | 1.136 | 0.070 |
| *Unknown* | 2.108 | 0.169 | 26.280 | 0.562 |
| Gender |  |  |  |  |
| *Male* | REFERENCE | | | |
| *Female* | 1.673 | 0.588 | 4.757 | 0.335 |
| Social Support |  |  |  |  |
| *Yes* | REFERENCE | | | |
| *No* | 0.378 | 0.059 | 2.418 | 0.304 |
| HD Vintage | 1.005 | 0.997 | 1.014 | 0.215 |
| Charlson Index | 1.204 | 0.913 | 1.586 | 0.188 |
| Cognitive Impairment |  |  |  |  |
| *No* | REFERENCE | | | |
| *Yes* | 0.258 | 0.061 | 1.097 | 0.067 |
| PHQ-9 Score | **1.147** | **1.032** | **1.275** | **0.011** |
| Self-Reported Health Change |  |  |  |  |
| *Better* | REFERENCE | | | |
| *The Same* | 1.664 | 0.383 | 7.236 | 0.497 |
| *Worse* | 0.814 | 0.209 | 3.178 | 0.767 |
| Self-Reported Health Today | 0.979 | 0.955 | 1.004 | 0.095 |
| Walking Aid Use |  |  |  |  |
| *No* | REFERENCE | | | |
| *Yes* | **6.657** | **2.435** | **18.199** | **<0.001** |
| Self-Reported Slow Walk Speed |  |  |  |  |
| *No* | REFERENCE | | | |
| *Yes* | **11.458** | **3.044** | **43.123** | **<0.001** |
| Constant | 0.271 | 0.004 | 18.611 | 0.545 |

Supplementary Table 8b: Multivariable logistic regression of Frailty Phenotype by Low Muscle Mass. Model 3

|  | Coefficient | Lower 95% C.I. | Upper 95% C.I. | P |
| --- | --- | --- | --- | --- |
| Low Muscle Mass |  |  |  |  |
| *No* | REFERENCE | | | |
| *Yes* | **4.160** | **1.356** | **12.765** | **0.013** |
| Age | 1.005 | 0.955 | 1.058 | 0.848 |
| Ethnicity |  |  |  |  |
| *White* | REFERENCE | | | |
| *South Asian* | 1.537 | 0.395 | 5.987 | 0.535 |
| *Black* | 0.515 | 0.125 | 2.126 | 0.359 |
| *Other* | OMITTED | | | |
| Education Level |  |  |  |  |
| *High School* | REFERENCE | | | |
| *College/6th form* | 0.633 | 0.207 | 1.943 | 0.424 |
| *University* | 0.100 | 0.007 | 1.503 | 0.096 |
| IMD Quintile |  |  |  |  |
| *1* | REFERENCE | | | |
| *2* | 1.392 | 0.343 | 5.650 | 0.643 |
| *3* | 0.558 | 0.148 | 2.099 | 0.388 |
| *4* | 0.138 | 0.018 | 1.080 | 0.059 |
| *5* | **0.150** | **0.025** | **0.882** | **0.036** |
| *Unknown* | 1.972 | 0.163 | 23.844 | 0.594 |
| Gender |  |  |  |  |
| *Male* | REFERENCE | | | |
| *Female* | **3.155** | **1.082** | **9.194** | **0.035** |
| Social Support |  |  |  |  |
| *Yes* | REFERENCE | | | |
| *No* | 0.308 | 0.046 | 2.064 | 0.225 |
| HD Vintage | 1.004 | 0.995 | 1.013 | 0.374 |
| Charlson Index | 1.225 | 0.932 | 1.609 | 0.146 |
| Cognitive Impairment |  |  |  |  |
| *No* | REFERENCE | | | |
| *Yes* | 0.245 | 0.057 | 1.060 | 0.060 |
| PHQ-9 Score | **1.153** | **1.038** | **1.282** | **0.008** |
| Self-Reported Health Change |  |  |  |  |
| *Better* | REFERENCE | | | |
| *The Same* | 1.629 | 0.371 | 7.154 | 0.518 |
| *Worse* | 0.813 | 0.210 | 3.146 | 0.765 |
| Self-Reported Health Today | 0.983 | 0.959 | 1.009 | 0.195 |
| Walking Aid Use |  |  |  |  |
| *No* | REFERENCE | | | |
| *Yes* | **6.219** | **2.232** | **17.326** | **<0.001** |
| Self-Reported Slow Walk Speed |  |  |  |  |
| *No* | REFERENCE | | | |
| *Yes* | **12.938** | **3.450** | **48.509** | **<0.001** |
| Constant | **0.011** | **0.000** | **0.504** | **0.021** |

Supplementary Table 8c: Multivariable logistic regression of Frailty Phenotype by Sarcopenia. Model 3

|  | OR | Lower 95% C.I. | Upper 95% C.I. | P |
| --- | --- | --- | --- | --- |
| Sarcopenia |  |  |  |  |
| *No* | REFERENCE | | | |
| *Yes* | **7.034** | **2.147** | **23.042** | **0.001** |
| Age | 0.991 | 0.939 | 1.046 | 0.756 |
| Ethnicity |  |  |  |  |
| *White* | REFERENCE | | | |
| *South Asian* | 1.145 | 0.276 | 4.743 | 0.852 |
| *Black* | 0.618 | 0.145 | 2.632 | 0.515 |
| *Other* | 1.000 |  |  |  |
| Education Level |  |  |  |  |
| *High School* | REFERENCE | | | |
| *College/6th form* | 0.517 | 0.168 | 1.585 | 0.248 |
| *University* | 0.085 | 0.005 | 1.440 | 0.088 |
| IMD Quintile |  |  |  |  |
| *1* | REFERENCE | | | |
| *2* | 1.816 | 0.463 | 7.119 | 0.392 |
| *3* | 1.006 | 0.268 | 3.769 | 0.993 |
| *4* | 0.191 | 0.024 | 1.543 | 0.120 |
| *5* | 0.190 | 0.030 | 1.221 | 0.080 |
| *Unknown* | 2.124 | 0.184 | 24.579 | 0.546 |
| Gender |  |  |  |  |
| *Male* | REFERENCE | | | |
| *Female* | 2.652 | 0.927 | 7.589 | 0.069 |
| Social Support |  |  |  |  |
| *Yes* | REFERENCE | | | |
| *No* | 0.225 | 0.029 | 1.738 | 0.153 |
| HD Vintage | 1.003 | 0.995 | 1.012 | 0.476 |
| Charlson Index | 1.227 | 0.925 | 1.627 | 0.156 |
| Cognitive Impairment |  |  |  |  |
| *No* | REFERENCE | | | |
| *Yes* | 0.241 | 0.051 | 1.145 | 0.073 |
| PHQ-9 Score | **1.144** | **1.029** | **1.271** | **0.013** |
| Self-Reported Health Change |  |  |  |  |
| *Better* | REFERENCE | | | |
| *The Same* | 1.533 | 0.328 | 7.156 | 0.587 |
| *Worse* | 0.966 | 0.240 | 3.893 | 0.961 |
| Self-Reported Health Today | 0.990 | 0.964 | 1.017 | 0.472 |
| Walking Aid Use |  |  |  |  |
| *No* | REFERENCE | | | |
| *Yes* | **8.121** | **2.790** | **23.642** | **<0.001** |
| Self-Reported Slow Walk Speed |  |  |  |  |
| *No* | REFERENCE | | | |
| *Yes* | **16.034** | **3.928** | **65.447** | **<0.001** |
| Constant | **0.016** | **0.000** | **0.812** | **0.039** |

Supplementary Table 9a: Multivariable logistic regression of Frailty Index by BATT. Model 3

|  | Coefficient | Lower 95% C.I. | Upper 95% C.I. | P |
| --- | --- | --- | --- | --- |
| BATT | 0.984 | 0.952 | 1.017 | 0.345 |
| Age | 1.014 | 0.968 | 1.063 | 0.554 |
| Ethnicity |  |  |  |  |
| *White* | REFERENCE | | | |
| *South Asian* | **5.488** | **1.171** | **25.714** | **0.031** |
| *Black* | 0.748 | 0.204 | 2.743 | 0.662 |
| *Other* | 2.680 | 0.118 | 60.795 | 0.536 |
| Education Level |  |  |  |  |
| *High School* | REFERENCE | | | |
| *College/6th form* | 1.302 | 0.402 | 4.220 | 0.660 |
| *University* | 1.178 | 0.253 | 5.492 | 0.835 |
| IMD Quintile |  |  |  |  |
| *1* | REFERENCE | | | |
| *2* | 0.733 | 0.197 | 2.724 | 0.643 |
| *3* | 2.041 | 0.518 | 8.050 | 0.308 |
| *4* | 0.289 | 0.054 | 1.542 | 0.146 |
| *5* | 0.650 | 0.159 | 2.651 | 0.548 |
| *Unknown* | 0.443 | 0.055 | 3.578 | 0.445 |
| Gender |  |  |  |  |
| *Male* | REFERENCE | | | |
| *Female* | 1.896 | 0.668 | 5.381 | 0.230 |
| Social Support |  |  |  |  |
| *Yes* | REFERENCE | | | |
| *No* | 0.408 | 0.074 | 2.252 | 0.304 |
| HD Vintage | 0.999 | 0.990 | 1.008 | 0.811 |
| Charlson Index | 1.106 | 0.833 | 1.468 | 0.485 |
| Cognitive Impairment |  |  |  |  |
| *No* | REFERENCE | | | |
| *Yes* | **8.406** | **2.450** | **28.837** | **0.001** |
| PHQ-9 Score | **1.294** | **1.137** | **1.473** | **<0.001** |
| Self-Reported Health Change |  |  |  |  |
| *Better* | REFERENCE | | | |
| *The Same* | 0.488 | 0.139 | 1.719 | 0.264 |
| *Worse* | 1.482 | 0.440 | 4.992 | 0.525 |
| Self-Reported Health Today | **0.965** | **0.942** | **0.989** | **0.005** |
| Walking Aid Use |  |  |  |  |
| *No* | REFERENCE | | | |
| *Yes* | **5.212** | **1.836** | **14.796** | **0.002** |
| Self-Reported Slow Walk Speed |  |  |  |  |
| *No* | REFERENCE | | | |
| *Yes* | 2.020 | 0.784 | 5.204 | 0.145 |
| Constant | 0.107 | 0.002 | 5.886 | 0.275 |

Supplementary Table 9b: Multivariable logistic regression of Frailty Index by Low Muscle Mass. Model 3

|  | Coefficient | Lower 95% C.I. | Upper 95% C.I. | P |
| --- | --- | --- | --- | --- |
| Low Muscle Mass |  |  |  |  |
| *No* | REFERENCE | | | |
| *Yes* | 0.971 | 0.357 | 2.639 | 0.955 |
| Age | 1.014 | 0.968 | 1.063 | 0.554 |
| Ethnicity |  |  |  |  |
| *White* | REFERENCE | | | |
| *South Asian* | **5.734** | **1.233** | **26.666** | **0.026** |
| *Black* | 0.788 | 0.217 | 2.856 | 0.717 |
| *Other* | 2.797 | 0.119 | 65.470 | 0.523 |
| Education Level |  |  |  |  |
| *High School* | REFERENCE | | | |
| *College/6th form* | 1.180 | 0.367 | 3.794 | 0.781 |
| *University* | 1.086 | 0.232 | 5.092 | 0.917 |
| IMD Quintile |  |  |  |  |
| *1* | REFERENCE | | | |
| *2* | 0.765 | 0.197 | 2.965 | 0.699 |
| *3* | 2.262 | 0.567 | 9.026 | 0.248 |
| *4* | 0.308 | 0.057 | 1.664 | 0.171 |
| *5* | 0.640 | 0.159 | 2.586 | 0.531 |
| *Unknown* | 0.468 | 0.057 | 3.801 | 0.477 |
| Gender |  |  |  |  |
| *Male* | REFERENCE | | | |
| *Female* | 2.202 | 0.813 | 5.961 | 0.120 |
| Social Support |  |  |  |  |
| *Yes* | REFERENCE | | | |
| *No* | 0.384 | 0.069 | 2.136 | 0.275 |
| HD Vintage | 1.000 | 0.991 | 1.009 | 0.954 |
| Charlson Index | 1.141 | 0.858 | 1.518 | 0.365 |
| Cognitive Impairment |  |  |  |  |
| *No* | REFERENCE | | | |
| *Yes* | **7.555** | **2.232** | **25.571** | **0.001** |
| PHQ-9 Score | **1.283** | **1.129** | **1.459** | **<0.001** |
| Self-Reported Health Change |  |  |  |  |
| *Better* | REFERENCE | | | |
| *The Same* | 0.489 | 0.139 | 1.718 | 0.264 |
| *Worse* | 1.504 | 0.447 | 5.058 | 0.509 |
| Self-Reported Health Today | **0.965** | **0.942** | **0.989** | **0.005** |
| Walking Aid Use |  |  |  |  |
| *No* | REFERENCE | | | |
| *Yes* | **5.576** | **1.924** | **16.162** | **0.002** |
| Self-Reported Slow Walk Speed |  |  |  |  |
| *No* | REFERENCE | | | |
| *Yes* | 2.124 | 0.832 | 5.421 | 0.115 |
| Constant | 0.042 | 0.001 | 1.396 | 0.076 |

Supplementary Table 9c: Multivariable logistic regression of Frailty Index by Sarcopenia. Model 3

|  | OR | Lower 95% C.I. | Upper 95% C.I. | P |
| --- | --- | --- | --- | --- |
| Sarcopenia |  |  |  |  |
| *No* | REFERENCE | | | |
| *Yes* | **3.033** | **1.038** | **8.862** | **0.043** |
| Age | 1.019 | 0.973 | 1.067 | 0.434 |
| Ethnicity |  |  |  |  |
| *White* | REFERENCE | | | |
| *South Asian* | **5.187** | **1.042** | **25.820** | **0.044** |
| *Black* | 0.827 | 0.226 | 3.023 | 0.774 |
| *Other* | 3.507 | 0.160 | 77.097 | 0.426 |
| Education Level |  |  |  |  |
| *High School* | REFERENCE | | | |
| *College/6th form* | 1.222 | 0.373 | 3.996 | 0.741 |
| *University* | 1.030 | 0.218 | 4.857 | 0.970 |
| IMD Quintile |  |  |  |  |
| *1* | REFERENCE | | | |
| *2* | 0.591 | 0.152 | 2.296 | 0.447 |
| *3* | 2.463 | 0.611 | 9.933 | 0.205 |
| *4* | 0.321 | 0.060 | 1.732 | 0.187 |
| *5* | 0.606 | 0.142 | 2.587 | 0.499 |
| *Unknown* | 0.426 | 0.051 | 3.524 | 0.428 |
| Gender |  |  |  |  |
| *Male* | REFERENCE | | | |
| *Female* | 2.344 | 0.847 | 6.487 | 0.101 |
| Social Support |  |  |  |  |
| *Yes* | REFERENCE | | | |
| *No* | 0.373 | 0.069 | 2.006 | 0.251 |
| HD Vintage | 0.998 | 0.989 | 1.007 | 0.679 |
| Charlson Index | 1.064 | 0.806 | 1.404 | 0.663 |
| Cognitive Impairment |  |  |  |  |
| *No* | REFERENCE | | | |
| *Yes* | **8.937** | **2.519** | **31.702** | **0.001** |
| PHQ-9 Score | **1.294** | **1.139** | **1.471** | **<0.001** |
| Self-Reported Health Change |  |  |  |  |
| *Better* | REFERENCE | | | |
| *The Same* | 0.425 | 0.116 | 1.561 | 0.198 |
| *Worse* | 1.434 | 0.417 | 4.926 | 0.567 |
| Self-Reported Health Today | **0.968** | **0.944** | **0.993** | **0.013** |
| Walking Aid Use |  |  |  |  |
| *No* | REFERENCE | | | |
| *Yes* | **5.501** | **1.896** | **15.962** | **0.002** |
| Self-Reported Slow Walk Speed |  |  |  |  |
| *No* | REFERENCE | | | |
| *Yes* | 2.302 | 0.881 | 6.014 | 0.089 |
| Constant | **0.025** | **0.001** | **0.905** | **0.044** |

Supplementary Table 10a: Multivariable logistic regression of Edmonton Frailty Scale by BATT. Model 3

|  | Coefficient | Lower 95% C.I. | Upper 95% C.I. | P |
| --- | --- | --- | --- | --- |
| BATT | **0.970** | **0.942** | **1.000** | **0.050** |
| Age | 0.987 | 0.947 | 1.030 | 0.558 |
| Ethnicity |  |  |  |  |
| *White* | REFERENCE | | | |
| *South Asian* | 1.802 | 0.586 | 5.546 | 0.304 |
| *Black* | **4.229** | **1.230** | **14.539** | **0.022** |
| *Other* | OMITTED | | | |
| Education Level |  |  |  |  |
| *High School* | REFERENCE | | | |
| *College/6th form* | 0.598 | 0.217 | 1.647 | 0.320 |
| *University* | 0.575 | 0.141 | 2.344 | 0.440 |
| IMD Quintile |  |  |  |  |
| *1* | REFERENCE | | | |
| *2* | 1.508 | 0.403 | 5.640 | 0.542 |
| *3* | 0.354 | 0.110 | 1.138 | 0.081 |
| *4* | 0.484 | 0.112 | 2.100 | 0.333 |
| *5* | 1.781 | 0.456 | 6.951 | 0.406 |
| *Unknown* | 0.642 | 0.131 | 3.158 | 0.586 |
| Gender |  |  |  |  |
| *Male* | REFERENCE | | | |
| *Female* | 1.035 | 0.422 | 2.541 | 0.940 |
| Social Support |  |  |  |  |
| *Yes* | REFERENCE | | | |
| *No* | 5.070 | 0.705 | 36.483 | 0.107 |
| HD Vintage | 0.994 | 0.987 | 1.002 | 0.144 |
| Charlson Index | 1.225 | 0.952 | 1.576 | 0.115 |
| Cognitive Impairment |  |  |  |  |
| *No* | REFERENCE | | | |
| *Yes* | 1.785 | 0.605 | 5.263 | 0.294 |
| PHQ-9 Score | **1.113** | **1.016** | **1.220** | **0.022** |
| Self-Reported Health Change |  |  |  |  |
| *Better* | REFERENCE | | | |
| *The Same* | 0.378 | 0.117 | 1.228 | 0.106 |
| *Worse* | 0.512 | 0.159 | 1.647 | 0.261 |
| Self-Reported Health Today | **0.972** | **0.952** | **0.994** | **0.012** |
| Walking Aid Use |  |  |  |  |
| *No* | REFERENCE | | | |
| *Yes* | **4.512** | **1.828** | **11.134** | **0.001** |
| Self-Reported Slow Walk Speed |  |  |  |  |
| *No* | REFERENCE | | | |
| *Yes* | 2.005 | 0.856 | 4.695 | 0.109 |
| Constant | 6.501 | 0.196 | 215.239 | 0.294 |

Supplementary Table 10b: Multivariable logistic regression of Edmonton Frailty Scale by Low Muscle Mass. Model 3

|  | Coefficient | Lower 95% C.I. | Upper 95% C.I. | P |
| --- | --- | --- | --- | --- |
| Low Muscle Mass |  |  |  |  |
| *No* | REFERENCE | | | |
| *Yes* | 2.165 | 0.878 | 5.336 | 0.093 |
| Age | 0.991 | 0.951 | 1.032 | 0.661 |
| Ethnicity |  |  |  |  |
| *White* | REFERENCE | | | |
| *South Asian* | 1.881 | 0.616 | 5.748 | 0.267 |
| *Black* | **4.101** | **1.218** | **13.811** | **0.023** |
| *Other* | OMITTED | | | |
| Education Level |  |  |  |  |
| *High School* | REFERENCE | | | |
| *College/6th form* | 0.629 | 0.226 | 1.751 | 0.374 |
| *University* | 0.583 | 0.145 | 2.349 | 0.448 |
| IMD Quintile |  |  |  |  |
| *1* | REFERENCE | | | |
| *2* | 1.247 | 0.329 | 4.724 | 0.745 |
| *3* | 0.344 | 0.104 | 1.136 | 0.080 |
| *4* | 0.487 | 0.109 | 2.167 | 0.345 |
| *5* | 1.553 | 0.405 | 5.953 | 0.520 |
| *Unknown* | 0.645 | 0.135 | 3.078 | 0.582 |
| Gender |  |  |  |  |
| *Male* | REFERENCE | | | |
| *Female* | 1.570 | 0.657 | 3.750 | 0.310 |
| Social Support |  |  |  |  |
| *Yes* | REFERENCE | | | |
| *No* | 5.565 | 0.714 | 43.364 | 0.101 |
| HD Vintage | 0.994 | 0.986 | 1.002 | 0.124 |
| Charlson Index | 1.231 | 0.959 | 1.580 | 0.102 |
| Cognitive Impairment |  |  |  |  |
| *No* | REFERENCE | | | |
| *Yes* | 1.761 | 0.600 | 5.165 | 0.303 |
| PHQ-9 Score | **1.108** | **1.014** | **1.212** | **0.024** |
| Self-Reported Health Change |  |  |  |  |
| *Better* | REFERENCE | | | |
| *The Same* | 0.364 | 0.114 | 1.169 | 0.090 |
| *Worse* | 0.547 | 0.173 | 1.726 | 0.303 |
| Self-Reported Health Today | **0.974** | **0.953** | **0.996** | **0.019** |
| Walking Aid Use |  |  |  |  |
| *No* | REFERENCE | | | |
| *Yes* | **4.201** | **1.702** | **10.368** | **0.002** |
| Self-Reported Slow Walk Speed |  |  |  |  |
| *No* | REFERENCE | | | |
| *Yes* | 2.201 | 0.937 | 5.168 | 0.070 |
| Constant | 0.621 | 0.033 | 11.815 | 0.751 |

Supplementary Table 10c: Multivariable logistic regression of Edmonton Frailty Scale by Sarcopenia. Model 3

|  | OR | Lower 95% C.I. | Upper 95% C.I. | P |
| --- | --- | --- | --- | --- |
| Sarcopenia |  |  |  |  |
| *No* | REFERENCE | | | |
| *Yes* | **3.238** | **1.207** | **8.681** | **0.020** |
| Age | 0.987 | 0.947 | 1.029 | 0.542 |
| Ethnicity |  |  |  |  |
| *White* | REFERENCE | | | |
| *South Asian* | 1.706 | 0.544 | 5.352 | 0.359 |
| *Black* | **4.131** | **1.221** | **13.975** | **0.023** |
| *Other* | 1.000 |  |  |  |
| Education Level |  |  |  |  |
| *High School* | REFERENCE | | | |
| *College/6th form* | 0.564 | 0.207 | 1.534 | 0.262 |
| *University* | 0.522 | 0.126 | 2.155 | 0.369 |
| IMD Quintile |  |  |  |  |
| *1* | REFERENCE | | | |
| *2* | 1.366 | 0.356 | 5.235 | 0.649 |
| *3* | 0.431 | 0.135 | 1.370 | 0.153 |
| *4* | 0.531 | 0.117 | 2.412 | 0.413 |
| *5* | 1.722 | 0.435 | 6.826 | 0.439 |
| *Unknown* | 0.641 | 0.132 | 3.126 | 0.583 |
| Gender |  |  |  |  |
| *Male* | REFERENCE | | | |
| *Female* | 1.480 | 0.625 | 3.508 | 0.373 |
| Social Support |  |  |  |  |
| *Yes* | REFERENCE | | | |
| *No* | 4.206 | 0.548 | 32.273 | 0.167 |
| HD Vintage | 0.993 | 0.986 | 1.001 | 0.101 |
| Charlson Index | 1.216 | 0.947 | 1.561 | 0.126 |
| Cognitive Impairment |  |  |  |  |
| *No* | REFERENCE | | | |
| *Yes* | 1.618 | 0.543 | 4.823 | 0.387 |
| PHQ-9 Score | **1.102** | **1.009** | **1.203** | **0.030** |
| Self-Reported Health Change |  |  |  |  |
| *Better* | REFERENCE | | | |
| *The Same* | 0.345 | 0.105 | 1.138 | 0.081 |
| *Worse* | 0.546 | 0.170 | 1.748 | 0.308 |
| Self-Reported Health Today | **0.977** | **0.955** | **0.999** | **0.037** |
| Walking Aid Use |  |  |  |  |
| *No* | REFERENCE | | | |
| *Yes* | **4.556** | **1.830** | **11.341** | **0.001** |
| Self-Reported Slow Walk Speed |  |  |  |  |
| *No* | REFERENCE | | | |
| *Yes* | 2.182 | 0.917 | 5.194 | 0.078 |
| Constant | 0.878 | 0.047 | 16.299 | 0.930 |

Supplementary Table 11a: Multivariable logistic regression of Clinical Frailty Scale by BATT. Model 3

|  | Coefficient | Lower 95% C.I. | Upper 95% C.I. | P |
| --- | --- | --- | --- | --- |
| BATT | 1.006 | 0.977 | 1.036 | 0.684 |
| Age | 1.005 | 0.963 | 1.048 | 0.831 |
| Ethnicity |  |  |  |  |
| *White* | REFERENCE | | | |
| *South Asian* | **3.647** | **1.026** | **12.961** | **0.046** |
| *Black* | 0.884 | 0.276 | 2.832 | 0.835 |
| *Other* | 2.638 | 0.152 | 45.912 | 0.506 |
| Education Level |  |  |  |  |
| *High School* | REFERENCE | | | |
| *College/6th form* | 1.012 | 0.362 | 2.827 | 0.982 |
| *University* | 2.306 | 0.599 | 8.875 | 0.225 |
| IMD Quintile |  |  |  |  |
| *1* | REFERENCE | | | |
| *2* | 2.558 | 0.779 | 8.399 | 0.122 |
| *3* | **3.743** | **1.089** | **12.867** | **0.036** |
| *4* | 0.433 | 0.090 | 2.077 | 0.295 |
| *5* | 1.015 | 0.262 | 3.932 | 0.983 |
| *Unknown* | 1.909 | 0.298 | 12.223 | 0.495 |
| Gender |  |  |  |  |
| *Male* | REFERENCE | | | |
| *Female* | **2.701** | **1.085** | **6.727** | **0.033** |
| Social Support |  |  |  |  |
| *Yes* | REFERENCE | | | |
| *No* | **0.147** | **0.030** | **0.725** | **0.019** |
| HD Vintage | 1.002 | 0.994 | 1.010 | 0.588 |
| Charlson Index | **1.322** | **1.008** | **1.733** | **0.044** |
| Cognitive Impairment |  |  |  |  |
| *No* | REFERENCE | | | |
| *Yes* | 2.937 | 0.971 | 8.885 | 0.056 |
| PHQ-9 Score | **1.144** | **1.045** | **1.252** | **0.004** |
| Self-Reported Health Change |  |  |  |  |
| *Better* | REFERENCE | | | |
| *The Same* | 0.727 | 0.233 | 2.270 | 0.583 |
| *Worse* | 0.949 | 0.315 | 2.862 | 0.926 |
| Self-Reported Health Today | 0.985 | 0.964 | 1.005 | 0.148 |
| Walking Aid Use |  |  |  |  |
| *No* | REFERENCE | | | |
| *Yes* | **6.370** | **2.557** | **15.871** | **<0.001** |
| Self-Reported Slow Walk Speed |  |  |  |  |
| *No* | REFERENCE | | | |
| *Yes* | **2.840** | **1.178** | **6.845** | **0.020** |
| Constant | **0.007** | **0.000** | **0.304** | **0.010** |

Supplementary Table 11b: Multivariable logistic regression of Clinical Frailty Scale by Low Muscle Mass. Model 3

|  | Coefficient | Lower 95% C.I. | Upper 95% C.I. | P |
| --- | --- | --- | --- | --- |
| Low Muscle Mass |  |  |  |  |
| *No* | REFERENCE | | | |
| *Yes* | 0.690 | 0.278 | 1.709 | 0.422 |
| Age | 1.003 | 0.962 | 1.047 | 0.876 |
| Ethnicity |  |  |  |  |
| *White* | REFERENCE | | | |
| *South Asian* | **3.677** | **1.032** | **13.101** | **0.045** |
| *Black* | 0.868 | 0.269 | 2.801 | 0.813 |
| *Other* | 2.576 | 0.143 | 46.387 | 0.521 |
| Education Level |  |  |  |  |
| *High School* | REFERENCE | | | |
| *College/6th form* | 0.972 | 0.344 | 2.749 | 0.957 |
| *University* | 2.219 | 0.567 | 8.692 | 0.252 |
| IMD Quintile |  |  |  |  |
| *1* | REFERENCE | | | |
| *2* | 2.854 | 0.838 | 9.717 | 0.093 |
| *3* | **3.988** | **1.147** | **13.864** | **0.030** |
| *4* | 0.437 | 0.090 | 2.114 | 0.303 |
| *5* | 1.060 | 0.272 | 4.131 | 0.933 |
| *Unknown* | 1.963 | 0.309 | 12.463 | 0.475 |
| Gender |  |  |  |  |
| *Male* | REFERENCE | | | |
| *Female* | 2.397 | 0.982 | 5.856 | 0.055 |
| Social Support |  |  |  |  |
| *Yes* | REFERENCE | | | |
| *No* | **0.146** | **0.030** | **0.719** | **0.018** |
| HD Vintage | 1.003 | 0.995 | 1.011 | 0.517 |
| Charlson Index | 1.332 | 1.017 | 1.746 | 0.038 |
| Cognitive Impairment |  |  |  |  |
| *No* | REFERENCE | | | |
| *Yes* | 2.925 | 0.966 | 8.862 | 0.058 |
| PHQ-9 Score | **1.141** | **1.042** | **1.249** | **0.004** |
| Self-Reported Health Change |  |  |  |  |
| *Better* | REFERENCE | | | |
| *The Same* | 0.737 | 0.235 | 2.315 | 0.601 |
| *Worse* | 0.937 | 0.309 | 2.835 | 0.908 |
| Self-Reported Health Today | 0.983 | 0.963 | 1.004 | 0.122 |
| Walking Aid Use |  |  |  |  |
| *No* | REFERENCE | | | |
| *Yes* | **6.712** | **2.652** | **16.989** | **<0.001** |
| Self-Reported Slow Walk Speed |  |  |  |  |
| *No* | REFERENCE | | | |
| *Yes* | **2.790** | **1.165** | **6.683** | **0.021** |
| Constant | **0.014** | **0.000** | **0.378** | **0.011** |

Supplementary Table 11c: Multivariable logistic regression of Clinical Frailty Scale by Sarcopenia. Model 3

|  | OR | Lower 95% C.I. | Upper 95% C.I. | P |
| --- | --- | --- | --- | --- |
| Sarcopenia |  |  |  |  |
| *No* | REFERENCE | | | |
| *Yes* | 1.533 | 0.600 | 3.919 | 0.372 |
| Age | 1.005 | 0.964 | 1.048 | 0.802 |
| Ethnicity |  |  |  |  |
| *White* | REFERENCE | | | |
| *South Asian* | 3.526 | 0.968 | 12.839 | 0.056 |
| *Black* | 0.950 | 0.296 | 3.057 | 0.932 |
| *Other* | 2.572 | 0.153 | 43.113 | 0.511 |
| Education Level |  |  |  |  |
| *High School* | REFERENCE | | | |
| *College/6th form* | 1.044 | 0.377 | 2.892 | 0.933 |
| *University* | 2.393 | 0.628 | 9.129 | 0.201 |
| IMD Quintile |  |  |  |  |
| *1* | REFERENCE | | | |
| *2* | 2.379 | 0.717 | 7.892 | 0.156 |
| *3* | **3.748** | **1.093** | **12.849** | **0.036** |
| *4* | 0.455 | 0.096 | 2.154 | 0.321 |
| *5* | 1.037 | 0.265 | 4.064 | 0.958 |
| *Unknown* | 1.824 | 0.283 | 11.780 | 0.528 |
| Gender |  |  |  |  |
| *Male* | REFERENCE | | | |
| *Female* | **2.641** | **1.094** | **6.375** | **0.031** |
| Social Support |  |  |  |  |
| *Yes* | REFERENCE | | | |
| *No* | **0.140** | **0.028** | **0.700** | **0.017** |
| HD Vintage | 1.001 | 0.994 | 1.010 | 0.715 |
| Charlson Index | 1.272 | 0.975 | 1.660 | 0.076 |
| Cognitive Impairment |  |  |  |  |
| *No* | REFERENCE | | | |
| *Yes* | **2.998** | **1.000** | **8.993** | **0.050** |
| PHQ-9 Score | **1.149** | **1.050** | **1.256** | **0.002** |
| Self-Reported Health Change |  |  |  |  |
| *Better* | REFERENCE | | | |
| *The Same* | 0.681 | 0.216 | 2.145 | 0.512 |
| *Worse* | 0.930 | 0.312 | 2.777 | 0.897 |
| Self-Reported Health Today | 0.988 | 0.967 | 1.009 | 0.252 |
| Walking Aid Use |  |  |  |  |
| *No* | REFERENCE | | | |
| *Yes* | **6.134** | **2.477** | **15.191** | **<0.001** |
| Self-Reported Slow Walk Speed |  |  |  |  |
| *No* | REFERENCE | | | |
| *Yes* | **2.839** | **1.180** | **6.829** | **0.020** |
| Constant | **0.009** | **0.000** | **0.242** | **0.005** |

Supplementary Table 12: Sensitivity Analyses of Frailty Score by multiple linear regression models in reverse order.

|  |  | Model | Coefficient | Lower 95% C.I. | Upper 95% C.I. | P |
| --- | --- | --- | --- | --- | --- | --- |
| Frailty Phenotype | BATT | A | **-0.013** | **-0.023** | **-0.003** | **0.009** |
|  |  | B | **-0.012** | **-0.022** | **-0.003** | **0.010** |
|  |  | C | -0.009 | -0.019 | 0.001 | 0.064 |
|  | LMM | A | **0.380** | **0.102** | **0.657** | **0.008** |
|  |  | B | **0.334** | **0.061** | **0.606** | **0.017** |
|  |  | C | **0.379** | **0.082** | **0.676** | **0.013** |
|  | Sarcopenia | A | **0.818** | **0.536** | **1.100** | **<0.001** |
|  |  | B | **0.740** | **0.448** | **1.032** | **<0.001** |
|  |  | C | **0.725** | **0.420** | **1.030** | **<0.001** |
| Frailty Index | BATT | A | 0.000 | -0.001 | 0.002 | 0.888 |
|  |  | B | 0.000 | -0.001 | 0.001 | 0.815 |
|  |  | C | 0.001 | -0.001 | 0.002 | 0.355 |
|  | LMM | A | -0.019 | -0.060 | 0.022 | 0.368 |
|  |  | B | -0.021 | -0.054 | 0.013 | 0.227 |
|  |  | C | -0.022 | -0.059 | 0.014 | 0.230 |
|  | Sarcopenia | A | **0.047** | **0.004** | **0.091** | **0.033** |
|  |  | B | 0.020 | -0.018 | 0.058 | 0.304 |
|  |  | C | 0.022 | -0.017 | 0.061 | 0.265 |
| Edmonton Frailty Scale | BATT | A | -0.012 | -0.034 | 0.011 | 0.300 |
|  |  | B | -0.015 | -0.036 | 0.006 | 0.151 |
|  |  | C | -0.020 | -0.042 | 0.001 | 0.064 |
|  | LMM | A | -0.031 | -0.683 | 0.620 | 0.924 |
|  |  | B | 0.125 | -0.481 | 0.732 | 0.684 |
|  |  | C | 0.277 | -0.383 | 0.938 | 0.408 |
|  | Sarcopenia | A | **0.840** | **0.150** | **1.530** | **0.017** |
|  |  | B | **0.951** | **0.283** | **1.618** | **0.005** |
|  |  | C | **1.101** | **0.411** | **1.791** | **0.002** |
| Clinical Frailty Scale | BATT | A | -0.004 | -0.015 | 0.007 | 0.493 |
|  |  | B | -0.003 | -0.014 | 0.008 | 0.597 |
|  |  | C | 0.001 | -0.011 | 0.012 | 0.912 |
|  | LMM | A | -0.033 | -0.346 | 0.281 | 0.838 |
|  |  | B | -0.084 | -0.400 | 0.232 | 0.601 |
|  |  | C | -0.074 | -0.416 | 0.269 | 0.672 |
|  | Sarcopenia | A | 0.293 | -0.042 | 0.627 | 0.086 |
|  |  | B | 0.113 | -0.241 | 0.467 | 0.530 |
|  |  | C | 0.104 | -0.263 | 0.471 | 0.575 |

Supplementary Table 13: Sensitivity analyses of multivariable logistic regression models of frailty. Multivariable models reversed.

|  |  | Model | OR | Lower 95% C.I. | Upper 95% C.I. | P |
| --- | --- | --- | --- | --- | --- | --- |
| Frailty Phenotype | BATT | A | **0.959** | **0.932** | **0.987** | **0.004** |
|  |  | B | **0.954** | **0.923** | **0.987** | **0.006** |
|  |  | C | 0.965 | 0.930 | 1.001 | 0.060 |
|  | LMM | A | **3.259** | **1.561** | **6.804** | **0.002** |
|  |  | B | **3.520** | **1.497** | **8.273** | **0.004** |
|  |  | C | **4.160** | **1.356** | **12.765** | **0.013** |
|  | Sarcopenia | A | **5.961** | **2.671** | **13.305** | **<0.001** |
|  |  | B | **6.334** | **2.434** | **16.484** | **<0.001** |
|  |  | C | **7.034** | **2.147** | **23.042** | **0.001** |
| Frailty Index | BATT | A | 0.992 | 0.971 | 1.014 | 0.491 |
|  |  | B | 0.981 | 0.953 | 1.010 | 0.192 |
|  |  | C | 0.984 | 0.952 | 1.017 | 0.345 |
|  | LMM | A | 0.891 | 0.469 | 1.694 | 0.725 |
|  |  | B | 1.023 | 0.442 | 2.367 | 0.957 |
|  |  | C | 0.971 | 0.357 | 2.639 | 0.955 |
|  | Sarcopenia | A | **2.426** | **1.181** | **4.985** | **0.016** |
|  |  | B | **2.722** | **1.035** | **7.160** | **0.042** |
|  |  | C | **3.033** | **1.038** | **8.862** | **0.043** |
| Edmonton Frailty Scale | BATT | A | 0.986 | 0.964 | 1.008 | 0.211 |
|  |  | B | 0.983 | 0.958 | 1.008 | 0.187 |
|  |  | C | 0.970 | 0.942 | 1.000 | 0.050 |
|  | LMM | A | -0.031 | -0.683 | 0.620 | 0.924 |
|  |  | B | 0.125 | -0.481 | 0.732 | 0.684 |
|  |  | C | 0.277 | -0.383 | 0.938 | 0.408 |
|  | Sarcopenia | A | **0.840** | **0.150** | **1.530** | **0.017** |
|  |  | B | **0.951** | **0.283** | **1.618** | **0.005** |
|  |  | C | **1.101** | **0.411** | **1.791** | **0.002** |
| Clinical Frailty Scale | BATT | A | 0.992 | 0.970 | 1.014 | 0.463 |
|  |  | B | 0.992 | 0.967 | 1.018 | 0.541 |
|  |  | C | 1.006 | 0.977 | 1.036 | 0.684 |
|  | LMM | A | 1.096 | 0.579 | 2.074 | 0.779 |
|  |  | B | 0.907 | 0.433 | 1.899 | 0.795 |
|  |  | C | 0.690 | 0.278 | 1.709 | 0.422 |
|  | Sarcopenia | A | **2.297** | **1.154** | **4.570** | **0.018** |
|  |  | B | 1.577 | 0.698 | 3.564 | 0.273 |
|  |  | C | 1.533 | 0.600 | 3.919 | 0.372 |

1. Fried LP, Tangen CM, Walston J, et al. Frailty in older adults: evidence for a phenotype. *J Gerontol A Biol Sci Med Sci*. Mar 2001;56(3):M146-56.

2. van Munster BC, Drost D, Kalf A, Vogtlander NP. Discriminative value of frailty screening instruments in end-stage renal disease. *Clin Kidney J*. Aug 2016;9(4):606-10. doi:10.1093/ckj/sfw061

3. Rolfson DB, Majumdar SR, Tsuyuki RT, Tahir A, Rockwood K. Validity and reliability of the Edmonton Frail Scale. *Age and Ageing*. September 1, 2006 2006;35(5):526-529. doi:10.1093/ageing/afl041
